# Reduced H3K27me3 leads to abnormal *Hox* gene expression in neural tube defects

**DOI:** 10.1186/s13072-019-0318-1

**Published:** 2019-12-19

**Authors:** Juan Yu, Lei Wang, Pei Pei, Xue Li, Jianxin Wu, Zhiyong Qiu, Juan Zhang, Ruifang Ao, Shan Wang, Ting Zhang, Jun Xie

**Affiliations:** 1 Department of Biochemistry and Molecular Biology, Shanxi Medical University, Taiyuan, 030001 Shanxi China; 2 Beijing Municipal Key Laboratory of Child Development and Nutriomics, Capital Institute of Pediatrics, Beijing, 100020 China; 3 Department of Biochemistry, Capital Institute of Pediatrics, Beijing, 100020 China; 4 School of Clinical Medical, Weifang Medical University, Weifang, 261053 Shandong China

**Keywords:** Neural tube defects, Retinoic acid, *Hox* genes, H3K27me3, UTX, SUZ12

## Abstract

**Background:**

Neural tube defects (NTDs) are severe, common birth defects that result from failure of normal neural tube closure during early embryogenesis. Accumulating strong evidence indicates that genetic factors contribute to NTDs etiology, among them, *HOX* genes play a key role in neural tube closure. Although abnormal *HOX* gene expression can lead to NTDs, the underlying pathological mechanisms have not fully been understood.

**Method:**

We detected that H3K27me3 and expression of the *Hox* genes in a retinoic acid (RA) induced mouse NTDs model on E8.5, E9.5 and E10.5 using RNA-sequencing and chromatin immunoprecipitation sequencing assays. Furthermore, we quantified 10 *Hox* genes using NanoString nCounter in brain tissue of fetuses with 39 NTDs patients including anencephaly, spina bifida, hydrocephaly and encephalocele.

**Results:**

Here, our results showed differential expression in 26 genes with a > 20-fold change in the level of expression, including 10 upregulated *Hox* genes. RT-qPCR revealed that these 10 *Hox* genes were all upregulated in RA-induced mouse NTDs as well as RA-treated embryonic stem cells (ESCs). Using ChIP-seq assays, we demonstrate that a decrease in H3K27me3 level upregulates the expression of *Hox* cluster *A–D* in RA-induced mouse NTDs model on E10.5. Interestingly, RA treatment led to attenuation of H3K27me3 due to cooperate between UTX and Suz12, affecting *Hox* gene regulation. Further analysis, in human anencephaly cases, upregulation of 10 *HOX* genes was observed, along with aberrant levels of H3K27me3. Notably, *HOXB4*, *HOX*C4 and *HOX*D1 expression was negatively correlated with H3K27me3 levels.

**Conclusion:**

Our results indicate that abnormal *HOX* gene expression induced by aberrant H3K27me3 levels may be a risk factor for NTDs and highlight the need for further analysis of genome-wide epigenetic modification in NTDs.

## Background

The neural tube is considered as the precursor of the future central nervous system (CNS) which comprises the brain and spinal cord [1]. Neural tube formation requires cell migration especially in the neural folds of the brain region, precise coordination between numerous cellular and molecular processes, and extensive interactions between the neural ectoderm, mesenchyme, and surface ectoderm across time and space. Multiple genes that regulate multiple cellular and molecular events need to work in concert, both temporally as well as spatially, for proper NT closure [2]. Failure to properly close the neural tube can result in neural tube defects (NTDs), the severity of which depends on the level of the body axis affected [3]. The etiologies of NTDs are complex and multifactorial; both genetic and environmental factors appear to be involved [4]. For example, imbalance of nutrient intake (e.g., folate or vitamin A) is an important risk factor during per-pregnancy. Vitamin A (all-trans retinol) and its active metabolites, collectively called retinoids, exert potent effects on stem cell differentiation and thus, the formation of the entire organism, in part via the modulation of the epigenome. However, how environmental factors affect the process of neural tube closure and their interaction with genetic factors remain largely unknown.

Recently, hundreds of genes have been shown to be regulated by RA during the processes of neuronal development. The *Hox* family includes four clusters, *Hoxa*, *b*, *c* and *d*, which encode transcriptional regulators that are highly conserved in vertebrates [5]. During embryogenesis, *Hox* genes exhibit temporal and spatial linearity of expression [6], and are essential for the anterior–posterior (head–tail) axis and neural tube development [7]. Although there is no direct evidence supporting the link between variants in *Hox* genes and NTDs in genetic studies [8]; however, it has been shown that aberrant expression of *Hox* genes can lead to NTDs [9, 10]. Recently, deregulation of gene expression through epigenetic mechanisms has been hypothesized to be a potential attribute for NTDs [11, 12] and results from recent studies indicate that *HOX* gene hypomethylation is a risk factor for NTDs [13].

Within chromatin, histone methylation regulates gene expression by either recruiting chromatin modifiers or directly altering chromatin structure, the biological consequence of which differs depending on the specific site where modifications occur [14]. Tri-methyl groups on lysine 27 of histone H3 (H3K27me3) induces gene silencing in cells, and such histone modification can pass through cell generations [14]. Genome-wide mapping has revealed H3K27me3 occupancy in a large set of genes related to cell fate and embryonic development, including developmental transcription factors (such as *Hox* genes) and cell-surface or extracellular proteins involved in cell fate regulation and patterning (such as Wnt [15]). These findings suggest that H3K27me3-regulated *Hox* gene expression might be associated with NTDs. H2K27me3 is critical mediator for transcription gene expression and contributes to important biological processes including animal body patterning. Our previous study indicated that folate deficiency attenuated H3K79me2, affecting some NTDs-associated genes, and interrupting early embryo development [16]. Enrichment of H3K4me1 at the hotspots of DSB regions enhances the recruitment of upstream binding factor to rRNA genes, resulting in the increase in transcription of rRNA genes [17]. It might provide the evidence that the risk of NTDs may be mediated through effecting histone methylation.

In this study, in an attempt to correlate the temporal and spatial expression pattern of *HOX* genes to NTDs, we employed a retinoic acid (RA)-induced mouse NTDs model. RNA-sequencing analysis produced detailed information on RA-induced transcriptome changes in the mouse embryo. Surprisingly, 26 genes were differentially expressed with levels changing over 20-fold, including 10 upregulated *Hox* genes. Using ChIP-seq assays, we demonstrate that a decrease in H3K27me3 level upregulates the expression of *Hox* cluster *A*–*D* in RA-induced mouse NTDs model on E10.5. Interestingly, RA treatment led to attenuation of H3K27me3 due to cooperate between UTX and Suz12, affecting *Hox* gene regulation. Further analysis, in human anencephaly cases, upregulation of 10 *HOX* genes was observed, along with aberrant levels of H3K27me3. Notably, *HOXB4*, *HOX*C4 and *HOX*D1 expression was negatively correlated with H3K27me3 levels. Taken together, our results provide the evidence that aberrant H3K27me3 levels is the link between abnormal *HOX* gene expression and NTDs, which further our understanding of the aberrant epigenetic modification of *Hox* genes in NTDs.

## Results

### A rapid RA-induced mouse NTDs model

RA, a derivative of vitamin A, is involved in neurulation and subsequent neural tube patterning, and plays an important role in mammalian development [18]. The association between vitamin A and birth defects comes from studies in which high doses were used. As a well-known teratogen, administration of RA to embryos induces NTDs, including spina bifida, exencephaly and anencephaly in several species. Given that a dose of RA of 28 mg/kg of body weight has been previously shown to cause significant anencephaly, we employed a modified rapid RA-induced NTDs mouse model via gavage of excess RA at E7.5 [19].

**Fig. 1:**
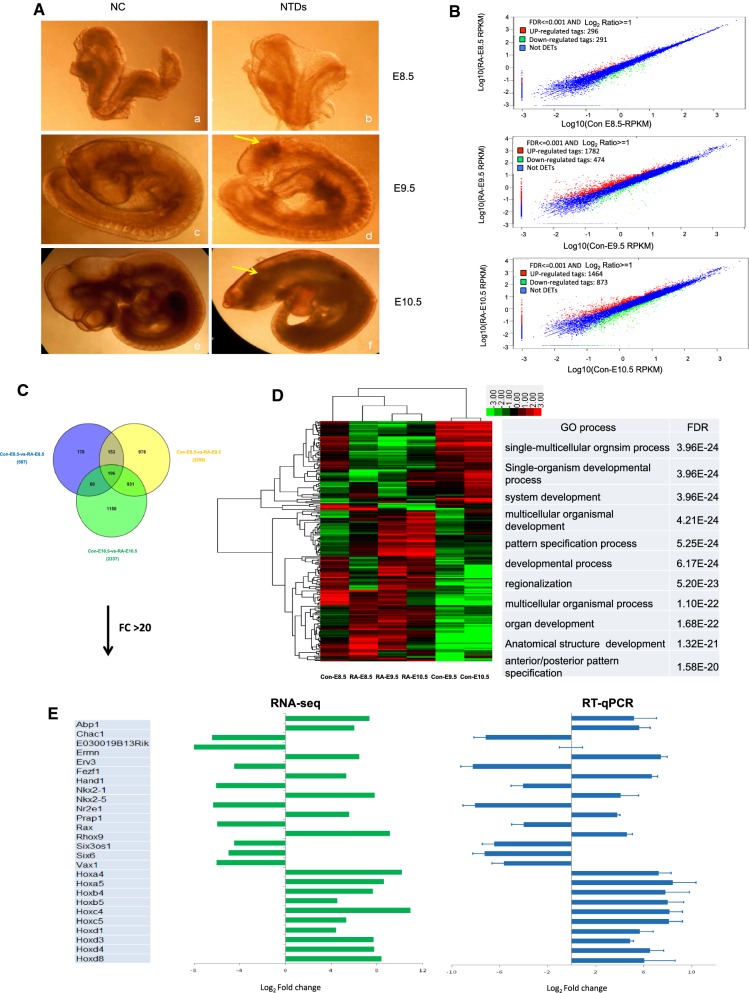
RA-induced mouse NTDs accompanied by dynamic transcriptome changes. **A** RA-induced anencephaly in C57BL/6 mouse embryos. a: Con-E8.5, b: RA-E8.5, c: Con-E9.5, d: RA-E9.5, e: Con-E10.5, f: RA-E10.5. Arrow indicated the abnormal section. **B** Differentially expressed genes (DEGs) identified in Con-E8.5-vs-RA-E8.5, Con-E9.5-vs-RA-E9.5 and Con-E10.5-vs-RA-E10.5 comparisons. **C** Intersection analysis of DEGs by Venny analysis. The overlaps represent the genes co-expressed in Con-E8.5-vs-RA-E8.5, Con-E9.5-vs-RA-E9.5 and Con-E10.5-vs-RA-E10.5 comparisons. **D** Hierarchical clustering plot showing representative expression patterns of 196 DEGs during mouse neural tube development. These genes were mainly classified into two categories. The top GO terms and corresponding enrichment *P* values are shown on the right side. **E** RT-qPCR validation of 26 DEGs identified by RNA-seq. *Actb* was used as control. Data are shown as the mean (SD; *n* = 3). *P* < 0.05 indicates statistical significance. Twenty five genes have a statistically significant change in expression but not the *Ermn* gene

Treatment with RA was overwhelmingly teratogenic and led to 94% of WT embryos showing defects with about two-thirds of the embryos showing anencephaly. As shown in Fig. 1A, morphological changes to the normal mouse embryo was prominent from E8.5 to E10.5 (Fig. 1A: a, c, e). After RA treatment, the morphology of mouse embryos differed significantly compared with that of control embryos. The defects were typical of anencephaly previously described following treatment with RA at this stage (Fig. 1A: b, d, f). On E8.5, RA-treated mouse embryos were similar qualitatively and quantitatively in the control embryos. On E9.5 and E10.5, RA-treated mouse embryos showed an anencephaly phenotype, maybe accompanied by growth retardation, enlarged heart and ventricular chambers, short tail, and unfinished turning of the neural axis [20] (Fig. 1A and Additional file 1: Figure S1A).

In both cell lines and embryos, RA target genes (e.g., *Hox* genes) are differentially induced by RA in a time- and RA concentration-dependent manner, where the genes at the 3′ end of the complexes are activated earlier and display the highest sensitivity to RA exposure [21]. It is hoped that understanding the molecular mechanisms of the RA response in cell culture will provide important insights into spatial colinearity in the embryo.

### Dynamic transcriptome changes and increased *Hox* gene expression induced by RA during neural tube development

To define alterations in gene expression profile accompanying RA-induced morphology changes, RNA-Seq was performed on mouse cranial tissue samples from E8.5, E9.5 and E10.5 embryos. Unsupervised clustering analysis revealed significant changes in transcriptome profile the time period between E8.5 and E10.5, during which morphological characteristics of NTDs became evident and accompany many genes changed (Additional file 1: Figure S1B). By comparing libraries from control and RA-induced samples at each of the three sample collecting time points, we identified a significant number of DEGs including 296 upregulated and 291 down-regulated genes on E8.5; 1782 upregulated and 474 down-regulated genes on E9.5; and 1464 upregulated and 873 down-regulated genes on E10.5, respectively (Fig. 1B). The majority of DEGs detected at each specific time point (E8.5, E9.5, E10.5) presented a respective distinctive profiling, however, certain degree of overlap of DEGs in samples from different times was observed (Fig. 1C). Further analysis with Gene Ontology (GO) indicated that these DEGs were enriched for GO terms of pattern specification, regionalization, cell differentiation and nervous system development and enriched in some pathways of glycolysis/gluconeogenesis, pathways in cancer, Hedgehog signaling pathway, etc. (Fig. 1D, Additional file 1: Figure S1C, D, Additional file 2: Table S1, Additional file 3: Table S2). Interestingly, variations in GO terms and DEG enrichment in a given pathway were observed between samples from different time points, suggesting the complex nature of NTDs at different development stage. In addition, PPI network suggested the complex regulating mechanisms in NTDs occurrence (Additional file 1: Figure S1E).

Overall, there were 196 DEGs with a greater than twofold change from E8.5 to E10.5. Of the 196 genes, 26 showed a greater than 20-fold change in expression which included 10 *Hox* genes that were strongly upregulated on E9.5 and E10.5. Results from RT-qPCR analysis of these 26 DEGs in cranial neural tissue of E10.5 embryos were consistent with the RNA-seq data (Fig. 1E). Taken together, our data suggest that gene expression pattern changes in RA-induced mouse NTDs and *Hox* genes may play key roles in this process.

### Elevation of *Hox* gene expression caused by reduced levels of H3K27me3 in RA-induced mouse NTDs

**Fig. 2:**
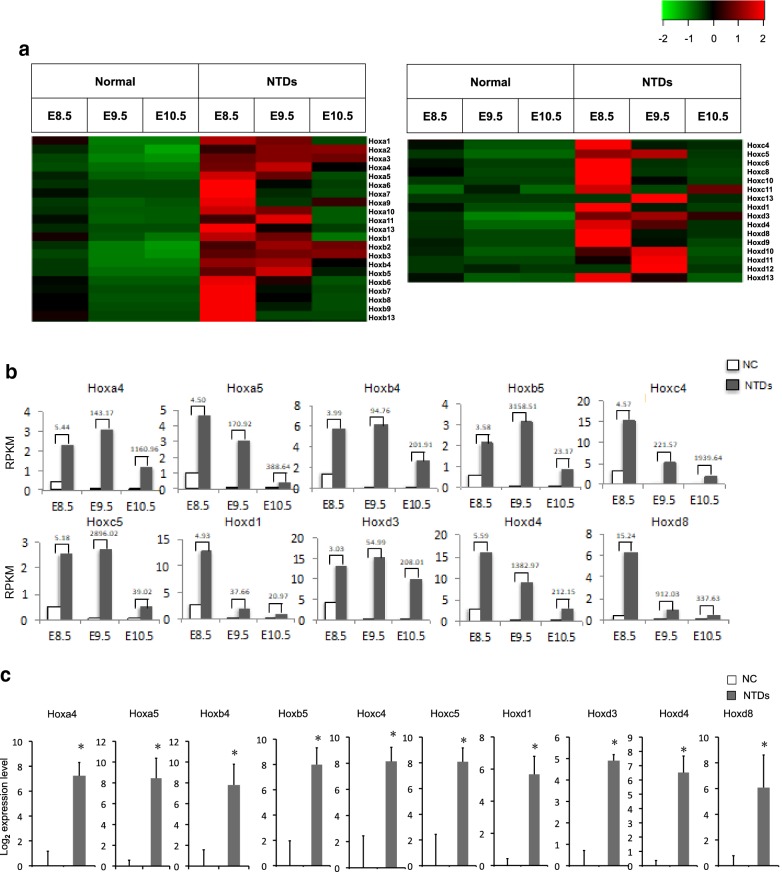
RA caused upregulation of *Hox* genes. **a** RNA-seq analysis of the dynamic expression of the four *Hox* clusters in cranial neural tissue of RA-induced mouse NTDs embryos from E8.5 to E10.5. **b** Dynamic expression of the 10 *Hox* genes in cranial neural tissue of RA-induced mouse NTDs embryos from E8.5 to E10.5. **c** *Hox* gene mRNA in cranial neural tissue of RA-induced mouse NTDs embryos was measured by RT-qPCR. *Actb* was used as control. Data are shown as the mean (SD; *n* = 3). *P < 0.05

During vertebrate embryogenesis, *Hox* genes exhibit temporal and spatial collinearity of expression, with the most centromeric *Hox* genes activated first and in the more anterior body structures, and the more telomeric *Hox* genes activated later and in the more posterior body structures [6]. To investigate the induction kinetics of *Hox* genes within each cluster in RA-induced mouse NTDs embryos with time, the expression of 37 *Hox* genes was compared between normal and RA-induced mouse NTDs embryos from E8.5 to E10.5. RNA-seq profiling revealed that the order of genes in the *Hox* clusters tended to temporally regulate, with a downward trending in expression in normal embryos (Fig. 2a). However, upon RA treatment, the *Hox* clusters displayed a significant upregulation at each developmental stage in mouse NTDs embryos from E8.5 to E10.5 (Fig. 2a). Since it was found that the expression of 10 *Hox* genes, including *Hoxa4*, *Hoxa5*, *Hoxb4*, *Hoxb5*, *Hoxc4*, *Hoxc5*, *Hoxd1*, *Hoxd3*, *Hoxd4*, and *Hoxd8*, was increased more than 20-fold in the RA-induced mouse NTDs embryos at E10.5 (Fig. 1E), we then evaluated the change in level of expression of these 10 *Hox* genes on E8.5, E9.5, and E10.5, respectively. In control embryos, the highest level of expression was seen on E8.5 for all 10 *Hox*s, with a downward trend (Fig. 2b). Upon RA-induction, upregulation of expression was evident in all 10 *Hox* genes, with the most prominent elevation in expression appeared on E9.5 or E10.5 (Fig. 2b). RT-qPCR assays were performed on cranial neural tissue of E10.5 mouse embryos, and the results indicated that levels of 10 *Hox* mRNA increased significantly, ranging between 29- and 347-fold, in NTDs embryos compared to that in controls (Fig. 2c). This further confirms that RA treatment upregulated expression of 10 selected *Hox* genes in mouse embryos, suggesting that *Hox* genes might play key roles in mouse early development.

#### Decrease of H3K27me3 accumulation at Hox loci in RA-induced mouse NTDs

**Fig. 3:**
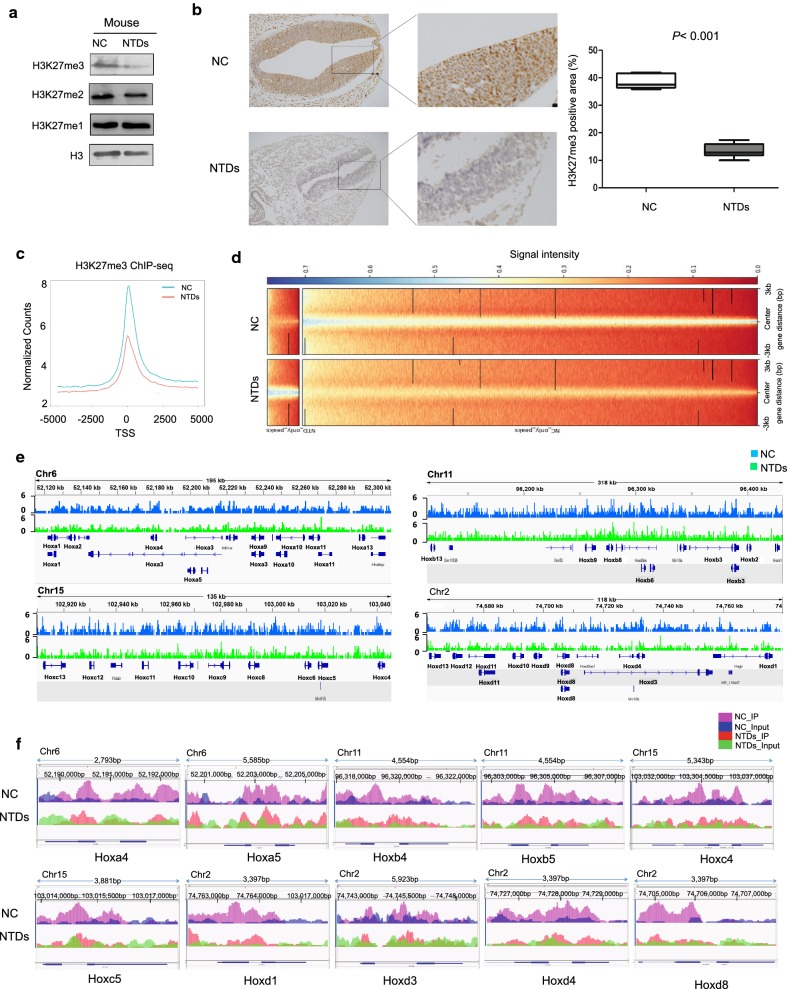
RA caused reduced levels of H3K27me3 at *Hox* loci in mouse NTDs embryos. **a** Cranial neural tissue of normal and RA-induced mouse NTDs was harvested at E10.5, and analyzed by western blotting. Numbers at the bottom were generated by quantification (ImageJ) of the H3K27me3/2/1 signal normalized to the H3 signal. **b** Immunohistochemistry staining was performed on transverse sections, for detecting H3K27me3 in NC and NTDs mouse embryos of E10.5. Scale bar: 50 μm. Areas of H3K27me3 positive cells were quantified by using ImageJ. Data are shown as the mean (SD; *n* = 5). *P < 0.05. **c** Comparison of average ChIP-Seq reads densities for H3K27me3 between NC and NTDs mouse embryos. **d** ChIP-Seq enrichment profiles for H3K27me3 levels in NC and NTDs mouse embryos. **e** ChIP-Seq density profiles for H3K27me3 at the mouse *Hox*a–*d* clusters in NC and NTDs mouse embryos. **f** ChIP-Seq density profiles for H3K27me3 at the 10 *Hox* genes in NC and NTDs mouse embryos

Previously data from genomic analysis indicate that the presence of H3K27me3 at transcriptional start sites is correlated with repression of *Hox* gene expression [7, 22]. We found that level of H3K27me3 and H3K27me2 was both decreased in RA-induced cranial neural tissue from E10.5 embryos, whereas no reduction was observed for H3K27me1 (Fig. 3a and Additional file 4: Figure S2A). Next, we also investigated whether the expression of H3K27me3 was abnormally altered in cranial neural tissue by immunohistochemical (IHC) analysis. To this end, we examined H3K27me3 expression levels in 3 pairs of RA-induced cranial neural tissue samples and their matched normal tissues by immunohistochemical analysis. The staining of total H3K27me3 decreased in mouse NTDs compared with that in their normal tissues (Fig. 3b).

To further explore the importance of histone H3K27me3 in neural tube defects, ChIP-seq was carried out in RA-induced mouse NTD model on E10.5. By analyzing equal numbers of reads from H3K27me3, a total of 52,759 and 13,059 peaks were detected in normal and NTDs mouse embryos, respectively, using an anti-H3K27me3 antibody, with the genome rate of 0.34% and 0.07% by scanning through the entire mouse genome (Additional file 4: Figure S2B). ChIP-seq of H3K27me3 target genes showed that the enrichment level of H3K27me3 was significantly decreased in RA-treated mouse on E10.5 compared with controls (Fig. 3c). By analyzing equal numbers of reads from H3K27me3, it also identified a remarkable reduction of H3K27me3 near transcription start sites in RA-treated mouse on E10.5 (Fig. 3d). Many peaks of H3K27me3 binding *Hoxa–d* genes clusters are decreased in RA-treated mouse on E10.5 (Fig. 3e). Consistent with this, we analyzed the specific region and accumulation of H3K27me3 peaks in 10 *Hox* genes (Fig. 3f). The result showed that accumulation of H3K27me3 in 10 *Hox* genes was reduced after RA treatment, which consistent with *Hox* clusters activation events. ChIP GO analysis showed that among genes targeted by H3K27me3 there was a bias toward genes related to the developmental process, especially nervous system development (Additional file 4: Figure S2C and Additional file 5: Table S3).

### Association of elevated *Hox* expression with depressed H3K27me3 in RA-induced mouse ESC

**Fig. 4:**
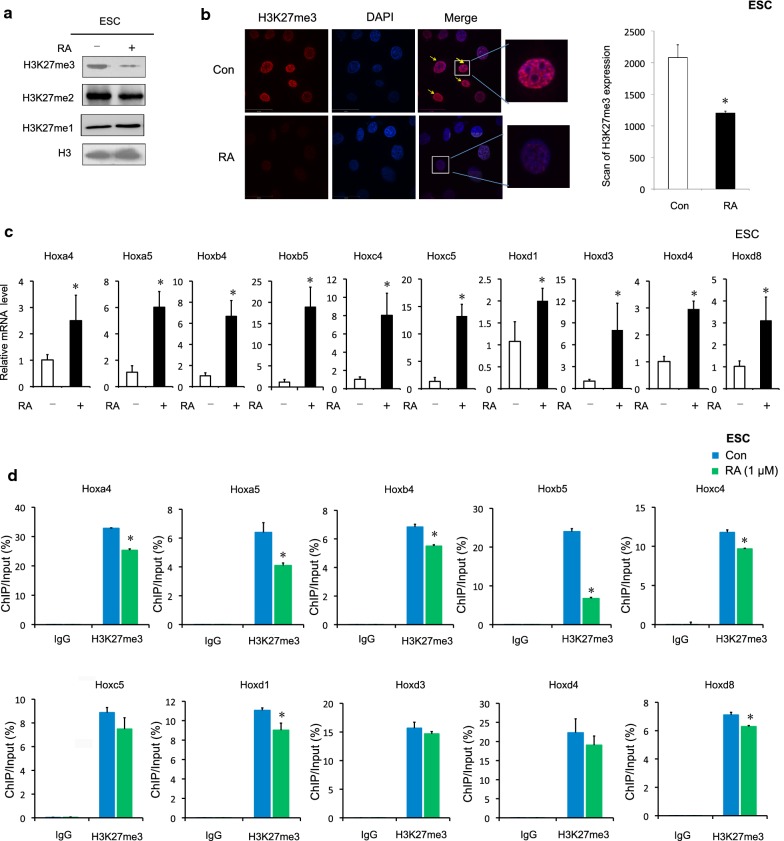
RA caused reduced levels of H3K27me3 at *Hox* genes in mouse ESCs. **a** Mouse ESCs were harvested after RA (1 μM) treatment for 24 h, and analyzed by western blotting. Numbers at the bottom were generated by quantification (ImageJ) of the H3K27me3/2/1 signal normalized to the H3 signal. **b** Immunostaining for H3K27me3 in RA-treated Hep-G2 cell. Direct immunofluorescence analysis was performed. Images were captured by confocal microscope and the nuclei were stained with DAPI. Scale bar: 35 μm. Data are shown as the mean (SD; *n* = 5). *P < 0.05. **c** *Hox* gene mRNA from mouse ESCs treated with RA was assessed by RT-qPCR. RA 1 μM, 24 h. *Actb* was used as control. Data are shown as mean (SD; *n* = 3). *P < 0.05. **d** ChIP assays of H3K27me3 were performed using mouse ESCs treated with 1 μM RA for 24 h. Mouse IgG was used as control. Enrichment of *Hox* gene promoters was measured by qPCR

Next, we detected that decrease in H3K27me3 and H3K27me2 level in RA-treated mouse ESCs, but no decrease in H3K27me1 level (Fig. 4a and Additional file 6: Figure S3A). Interestingly, it is likely that RA efficiently reduced the levels of H3K27me3 > H3K27me2. Immunofluorescence staining for H3K27me3 indicated that the loss of H3K27me3 in RA-treated cells. H3K27me3-enriched foci appeared to be localized to dense heterochromatic chromocenters in control cells, while the H3K27me3 immunostaining distribution was largely diffuse or speckled, with decreased signal intensity in RA-treated cells (Fig. 4b). In addition, RA treatment also led to an increase in the levels of all 10 selected *Hox* gene mRNAs in mouse ESCs (*P *< 0.05) (Fig. 4c). Besides, chromatin immunoprecipitation (ChIP) assays were performed on mouse ESCs to evaluate the enrichment of H3K27me3 to the selected 10 *Hox* genes. As shown in Fig. 4d, enrichment of H3K27me3 in these 7 *Hox* genes (*Hoxa4*, *Hoxa5*, *Hoxb4*, *Hoxb5*, *Hoxc4*, *Hoxd1* and *Hoxd8*) sequences was significant attenuated after RA treatment. The most significant attenuation was observed in enrichment of H3K27me3 in *Hoxb*5 for which a 72% decrease in the union was observed (Fig. 4d). By contrast, no change in enrichment of H3K27me3 in IgG loci sequence was observed (Fig. 4d). To further verify the effects of H3K27me2 enrichment on the Hox genes upon RA treatment, we also performed quantitative ChIP assays. The results showed that RA has no significant effects H3K27me2 enrichment on the sequences of most of Hox genes (Additional file 6: Figure S3B) in mouse ESCs. It is possible that H3K27me2 recruited to specific RA-inducible Hox genes. Collectively, our data indicate that during RA treatment, the enrichment of H3K27me3 on *Hox* genes (*Hoxa4*, *Hoxa5*, *Hoxb4*, *Hoxb5*, *Hoxc4*, *Hoxd1* and *Hoxd8*) were decreased, their expression level increased, and the overall level of histone H3K27me3 was decreased.

### UTX activity is important for RA-induced *Hox* upregulation

**Fig. 5:**
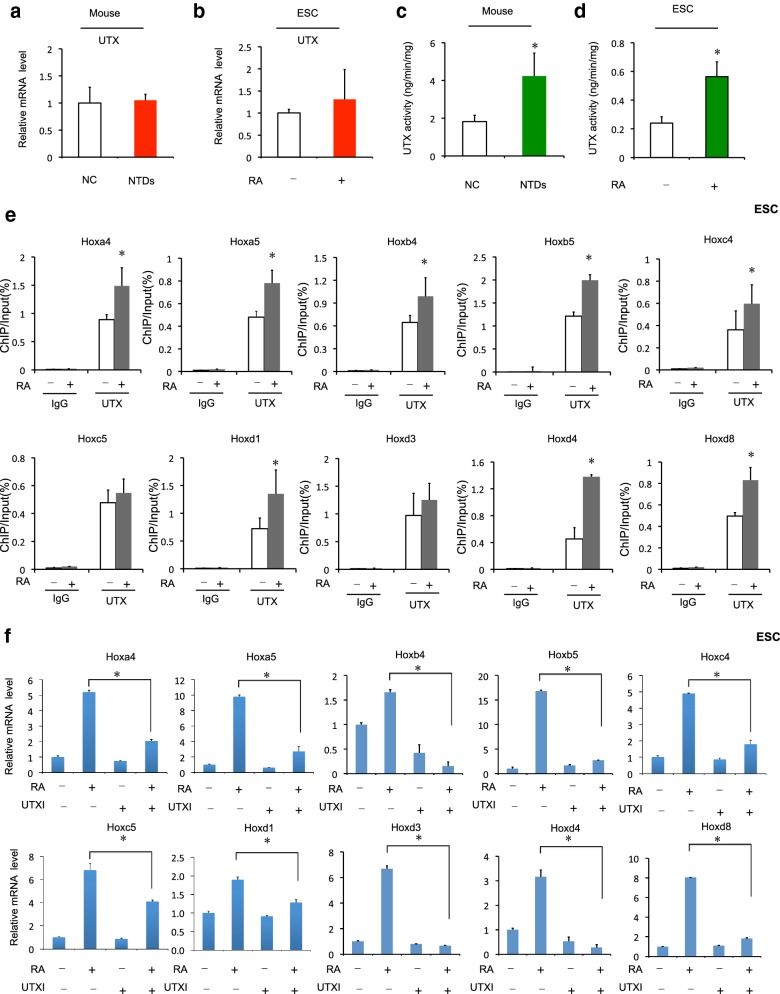
UTX activity plays the important role in RA-induced *Hox* upregulation. **a** UTX mRNA in cranial neural tissue of RA-induced mouse NTDs was measured by RT-qPCR. *Actb* was used as a loading control. Data are shown as the mean (SD; *n* = 4). *P < 0.05. **b** UTX mRNA in mouse ESCs treated with RA was measured by RT-qPCR. *Actb* was used as a loading control. Data are shown as the mean (SD; *n* = 3). *P < 0.05. **c** UTX demethylase activity was detected in cranial neural tissue of RA-induced NTD mouse embryos. Data are shown as the mean (SD; *n* = 4). *P < 0.05. **d** UTX demethylase activity was detected in mouse ESCs after RA treatment. Data are shown as the mean (SD; *n* = 3). *P < 0.05. **e** ChIP assays of UTX were performed using mouse ESCs treated with 1 μM RA for 24 h. Mouse IgG was used as control. Enrichment of *Hox* gene promoters was measured by qPCR. **f** GSK-J4 (UTX inhibitor) affected mRNA levels of *Hox* genes in RA-induced mouse ESCs. Mouse ESCs were treated with GSK-J4 (30 nM) for 6 h. Then, after 24 h of RA treatment, cells were collected and analyzed. Data are shown as the mean (SD; *n* = 3)

Previously, it has been shown that UTX demethylates H3K27me3 at the *Hox* loci, controls posterior development of zebrafish [23]. We were interested to explore the possibility that the decrease level of H3K27me3 in RA-induced mouse NTDs embryos and RA-treated mouse ESCs was due to the action of UTX, a key factor for embryonic development. No significant difference in level of expression was observed between samples from normal and NTDs embryos (Fig. 5a). Equivalent results were obtained in RA-induced mouse ESCs (Fig. 5b). Interestingly, the demethylase activity of UTX, which is capable of removing the methyl groups from H3K27me3, increased in RA-induced NTDs mouse cranial tissue and RA-treated ESCs (Fig. 5c, d).

Next, we examined the binding of UTX to the promoters of the selected 10 *Hox* genes using ChIP-qPCR assay. As shown in Fig. 5e, while no changes were seen for the binding to UTX to promoter regions of IgG, significantly increased UTX binding to the promoter regions of the *Hoxb5* and *Hoxd4* genes were evident upon RA treatment in mouse ESCs. Thus, RA treatment led to increased UTX activity, demethylation of H3K27me3, and subsequently an attenuation of H3K27me3 enrichment to the promoters of *Hox* genes.

To further confirm that an increase in UTX activity upon RA treatment was responsible for the increase in the expression of the selected *Hox* genes, we utilized a potent UTX inhibitor, GSK-J4, in our experiment. GSK-J4 treatment resulted in *Hoxa4*, *Hoxa5*, *Hoxb4*, *Hoxc4*, *Hoxc5*, *Hoxd4* genes were down-regulated significantly. However, *Hoxb5*, *Hoxd1*, *Hoxd3*, *Hoxd8* were slightly change but no significantly in UTX inhibitor-treated mouse ESCs (line 1 vs line 3). We next investigated whether UTX demethylase is involved in the RA-induced reduction of H3K27me3 levels at *Hox* genes in mouse ESCs. The expression of the *Hox* genes was increased in RA-treated mouse ESCs (line 1 vs line 2), but was much less RA-induced in UTX inhibitor-treated mouse ESCs (line 2 vs line 4) (Fig. 5f). Taken together, these data provide evidence indicating that RA treatment of mouse embryos and ESCs causes a decrease H3K27me3 methylation due to an increased demethylase activity of UTX. Recruitment of UTX to *Hox* promoters coincides with disappearance of H3K27me3, and *Hox* gene activation.

### Suz12 is required for RA-induced *Hox* upregulation

**Fig. 6:**
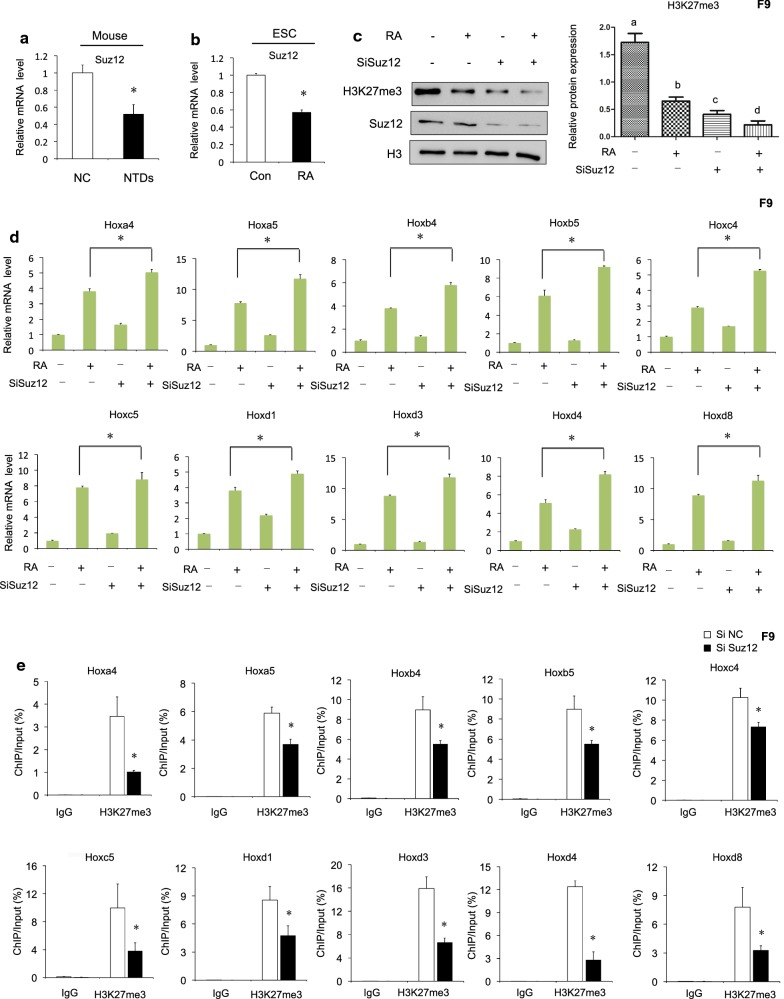
Suz12 is required for RA-induced *Hox* upregulation. **a** Suz12 mRNA in cranial neural tissue of RA-induced mouse NTDs was measured by RT-qPCR. *Actb* was used as a loading control. Data are shown as the mean (SD; *n* = 4). *P < 0.05. **b** Suz12 mRNA in mouse ESCs treated with RA was measured by RT-qPCR. *Actb* was used as a loading control. Data are shown as the mean (SD; *n* = 3). *P < 0.05. **c** H3K27me3 level after upon RA treatment was measured by Western blotting, respectively, by knockdown of Suz12. F9 cells were knockdown of Suz12 for 24 h, and then treated with RA. H3 was used as a loading control. **d** Knockdown of Suz12 affected mRNA level of *Hox* genes in RA-induced F9 cells. F9 cells were knockdown of Suz12 for 24 h. Then, after 24 h of RA treatment, cells were collected and analyzed. Data were shown as mean ± SD (*n* = 3). *P < 0.05. **e** ChIP assays of H3K27me3 were performed using mouse F9 cells after siSuz12 transfection. Mouse IgG was used as control. Enrichment of *Hox* gene promoters was measured by qPCR

Suz12 is physically associated with EZH2 and enhances H3K27me3 at its target genes. It is essential for polycomb repressive complex 2 (PRC2) activity and is required for embryonic stem cell differentiation, and which inactivation results in early lethality and NTDs occurence of mouse embryos [24]. In our study, the level of Suz12 expression was decreased significantly in NTDs embryos (Fig. 6a). RNA-seq analysis showed that the Suz12 gene expression was lower in NTDs embryos on E9.5 and E10.5 (Additional file 7: Figure S4A). Equivalent results were obtained in RA-induced mouse ESCs (Fig. 6b). In addition, Ezh2 expression was also decreased in RA-induced mouse NTDs embryos and mouse ESCs (Additional file 7: Figure S4B–D). We next investigated H3K27me3 by downregulating Suz12 under RA treatment. Level of H3K27me3 was decreased upon Suz12 depletion (line 1 vs line 3). Furthermore, depletion of Suz12 significantly decreased H3K27me3 after RA treatment (Fig. 6c). And, overexpression of Suz12 rescued the RA-reduced in the expression of the H3K27me3 (Additional file 7: Figure S4C). We next investigated whether Suz12 is involved in the RA-induced *Hox* genes in F9 cells. *Hox* genes were upregulated in Suz12-depleted F9 cells (line 1 vs line 3). We next investigated whether Suz12 is involved in the reduction of H3K27me3 levels *Hox* genes in RA-treated F9 cells. The inhibition of Suz12 expression by siRNA led to a strong increase in the levels of mRNAs encoded by *Hox* genes both before and after RA treatment (Fig. 6d). ChIP assay showed H3K27me3 enrichment in these 10 *Hox* genes was significant decreased after knockdown of Suz12 and responsible for the increase in the expression of the *Hox* genes. By contrast, no enrichment of H3K27me3 in IgG loci was observed (Fig. 6e). These data indicated that RA treatment of mouse embryos and ESCs could also cause a decrease H3K27me3 methylation through depressing Suz12, and led to *Hox* genes activation. Taken together, these findings provide evidence indicating that Suz12 effect on level of H3K27me3 at *Hox* genes.

### Negative correlation of *HOXB4*, *HOX*C4 and *HOX*D1 expression with H3K27me3 in human anencephaly

**Fig. 7:**
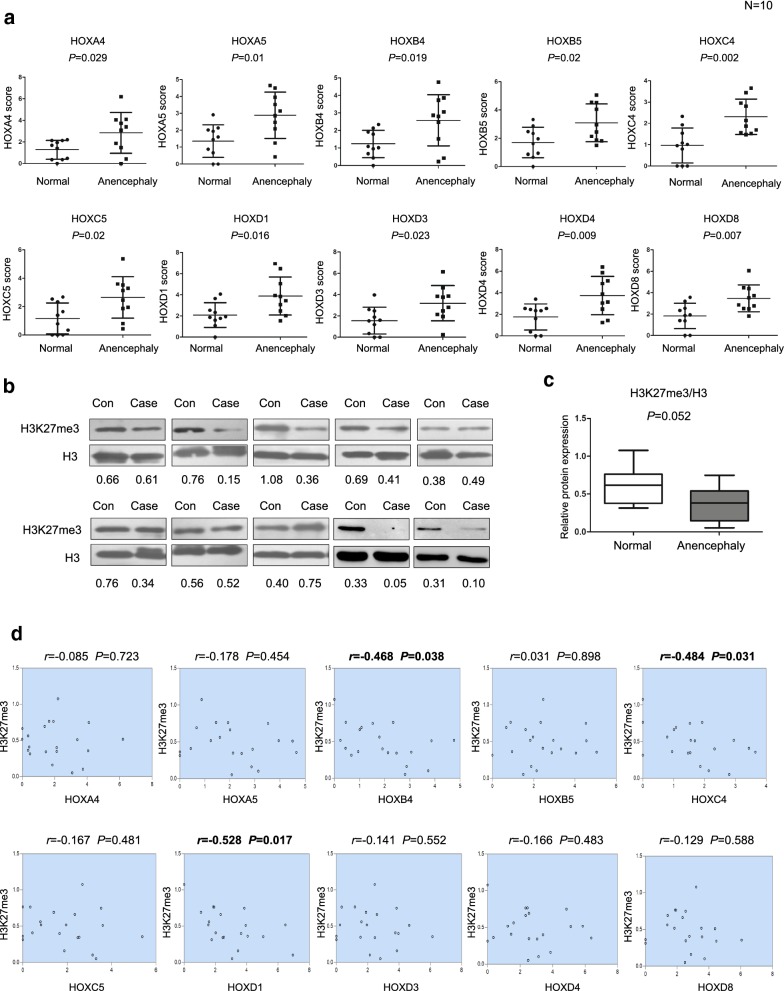
*HOX* gene expression was upregulated and H3K27me3 levels were decreased in human anencephaly. **a** *HOX* genes were detected with NanoString in the brain tissues from human anencephaly and normal cases. Data are shown as the mean (SD; *n* = 10). *P* < 0.05 indicates statistical significance. **b** Detection of histone H3K27me3 modification in brain tissues from human anencephaly and normal cases by western blotting. Total histone H3 was used as a loading control. Data are shown as the mean (SD; *n* = 10). **c** Quantification analysis of the H3K27me3 signal normalized to the H3 signal between human anencephaly and normal cases. **d** Pearson’s correlation analysis between *HOX*s expression and H3K27me3 level. *P* < 0.05 indicates statistical significance

*HOX* genes encode highly conserved transcription factors expressed in the brain and spinal cord that play a central role in establishing the anterior–posterior body axis during embryogenesis. Their expression is tightly regulated in a spatiotemporal and collinear manner, partly by chromatin structure and epigenetic modifications. To investigate the potential clinical relevance of expression levels of the selected *HOX* genes in anencephaly, we first examined the mRNA level of *HOX* genes, including *HOXA4*, *HOXA5*, *HOXB4*, *HOXB5*, *HOXC4*, *HOXC5*, *HOXD1*, *HOXD3*, *HOXD4*, and *HOXD8* in brain tissues from 39 NTD-affected and 39 normal control human fetuses. The clinical phenotypes of the cases were 10 anencephaly, 10 spina bifida, 10 spina bifida combined with hydrocephaly and 9 encephalocele. NanoString assays showed that expression of the 10 *HOX* genes (*HOXA4*, *HOXA5*, *HOXB4*, *HOXB5*, *HOXC4*, *HOXC5*, *HOXD1*, *HOXD3*, *HOXD4*, and *HOXD8*) was significantly upregulated in anencephaly tissues compared with normal tissues (*P* < 0.05) (Fig. 7a and Table 1). However, the results showed that in spina bifida, hydrocephaly and encephalocele level of 10 *HOX* genes were no significantly increased (Additional file 8: Figure S5A–C). Western blot analysis of ten anencephaly subjects compared with age- and gender-matched controls revealed that H3K27me3 levels decreased obviously in six anencephaly samples (Fig. 7b, c). The average level of H3K27me3 expression (normalized to H3) was found to be significantly lower in NTDs samples (*P *= 0.052). Interestingly, genetic studies could not show an association between variants in *HOX* genes and NTDs. We sequenced in 163 stillborn or miscarried with NTDs. The number of variants by minor allele frequency (MAF) and impact group in NTDs sample (Additional file 8: Figure S5D–I). The relative expression of H3K27me3 was decreased 30%. Importantly, significantly negative correlations of *HOXB4*, *HOXC4* and *HOXD1* expression were observed with H3K27me3 levels among the examined subjects (*r*_HOXB4_= − 0.468, *P *= 0.038; *r*_HOXC4_= − 0.484, *P *= 0.031; *r*_HOXD1_ = − 0.528, *P *= 0.017) (Fig. 7d). In conclusion, these data identify the abnormal upregulation of *HOX* genes, especially *HOXB4, HOXC4* and *HOXD1*, concomitant with decreased H3K27me3 levels in human anencephaly cases. This work is the first to demonstrate that in NTDs, especially anencephaly, increased *HOX* gene expression was accompanied by aberrant H3K27me3 levels.

**Table 1:** mRNA expression of 10 *HOX* genes in human anencephaly

Gene	Con ($\bar{X} \pm S$)	NTDs ($\bar{X} \pm S$)	*t*	*P* value
*HOXA4*	1.270 ± 0.867	2.835 ± 1.891	− 2.379	0.029
5*HOXA5*	1.357 ± 0.956	2.887 ± 1.368	− 2.899	0.010
*HOXB4*	1.234 ± 0.774	2.577 ± 1.463	− 2.567	0.019
*HOXB5*	1.705 ± 1.069	3.090 ± 1.334	− 2.562	0.020
*HOXC4*	0.963 ± 0.814	2.311 ± 0.831	− 3.664	0.002
*HOXC5*	1.174 ± 1.093	1.650 ± 1.459	− 2.561	0.020
*HOXD1*	2.085 ± 1.175	3.886 ± 1.800	− 2.650	0.016
*HOXD3*	1.599 ± 1.261	3.190 ± 1.638	− 2.496	0.023
*HOXD4*	1.759 ± 1.213	3.744 ± 1.781	− 2.913	0.009
*HOXD8*	1.822 ± 1.191	3.466 ± 1.249	− 3.012	0.007

Data were shown as mean ± SD (*n* = 10). *P* < 0.05 means the difference have statistical significance

## Discussion

Neural tube defects (NTDs) are multifactorial disorders that arise from a combination of genetic and environmental interactions. Many studies have focused on screening for candidate NTD genes, leading to the discovery of more than 200 genes known to cause NTDs in mouse [25, 26], although few of these have been validated in human NTDs. Results from recent studies show that not only gene mutations but also aberrant gene expression are important in NTDs [27]. Previous work from our laboratory has confirmed that abnormal expression of Wnt2b and Wnt7b is involved in human NTDs [28]. In this study, we attempted to explore the role of abnormal expression of *HOX* genes in NTDs as well as its underlining regulatory mechanism, based our data from transcriptome profile analysis of RA-induced mouse NTDs embryos.

The clustered *Hox* genes are required for establishing the embryonic anterior–posterior axis, and are important for embryogenesis. A unique feature of the clustered *Hox* genes is the direct relationship between their expression and function in time and space during development, termed collinearity, disruption of which can result in abnormal neural tube closure [29, 30]. Each NTDs phenotype depends on a different set of genes, such as *Hox* genes, which are activated in a temporally collinear manner to drive the progressive specification of different segments. Our RNA-seq data from this study showed the expression of 10 *Hox* genes decreased during mouse neural tube development, consistent with what has been reported from a previous study [31]. However, in RA-treated mouse embryo, all of these genes were significantly upregulated. In our study, we selected a window in time during development critical for normal neuronal tube closure, between E8.5 and E10.5. In normal mouse embryo, we observed a downward trending for the temporal expression of all *Hox* genes, from E8.5 to E10.5. On E8.5, the difference in level expression of *Hox* genes between control and RA-induced was insignificant, however, a drastic increase in *Hox* gene expression was observed on E9.5 and 10.5. Therefore, we reasoned that the accumulative disruption of the normal *Hox* gene expression level on E8.5 through E10.5 may have a profound effect of developmental processes leading to NTDs.

Components of histone modification have been shown recently to be critical for normal brain function and development, and aberrant levels of modification in these components contribute to nervous system diseases [32, 33]. Our previous data have also established associations between histone modifications and DNA methylation in human NTDs [34, 35]. Among all NTD-related histone modification components, a direct link between abnormal level of H3K27me3 and upregulation of *Wnt* genes has been demonstrated in NTDs [28]. In the present study, decreased levels of H3K27me3 were found in both RA-induced mouse anencephaly and sample from a number of human anencephaly patients. In order to explore the possibility whether the decreased level of H3K27me3 is associated with abnormal *Hox* gene expression, we employed ChIP-qPCR on samples from RA-treated mouse ESCs to evaluate the enrichment of H3K27me3 in *Hox* genes. Indeed, data from ChIP-qPCR revealed that enrichment of H3K27me3 was decreased in all 10 *Hox* genes, suggesting that *Hox* upregulation was caused by decreased H3K27me3 levels. In addition, mutation screens eliminated the possibility that specific mutations on *HOX* genes may occur that causes a diminished enrichment of H3K27me3 in the *HOX* genes in Chinese NTDs. Lastly, we evaluated the expression and activity of H3K27 demethylases in RA-treated samples since it direct affects H3K27me3 levels. The expression level of UTX, the enzyme demethylates H3K27 at the *Hox* loci, was unchanged during RA treatment. However, RA treatment led to an increase in UTX enzymatic activities, enabling elevated demethylating of H3K27me3. Previous results show that UTX binding correlates with diminished levels of H3K27me3 at transcription start sites [23]. Active regions of the *Hox* cluster are marked by long, continuous H3K4me2/3 stretches devoid of H3K27me3. UTX is not the only H3K27 demethylase throughout this long stretch. For example, another H3K27 demethylase, JMJD3, is associated with the *Hox*a7 and *Hox*a11 loci during bone marrow cell differentiation [32]. This suggests multiple H3K27 demethylases control expression of *Hox* gene clusters and it is tempting to speculate that additional H3K27 demethylases involved in transcriptional repression could more effectively regulate transcription [36].

One of the most intriguing questions remaining is that in our study, *HOX* gene expression was only increased in human anencephaly cases, but was not increased in encephalocele or spina bifida cases. Encephalocele is a phenotype of NTD that is different from anencephaly, although the lesion is located in the brain, while the spina bifida lesion is in the spinal cord. In this study, we collected brain tissue from human fetuses with different NTDs; therefore, it is not surprising that *HOX* genes were expressed differently in the brain in the different NTDs phenotypes. This result indicated the NTDs are not one disease, but many; therefore, it is better to explore the etiology according to the NTDs phenotype. A limitation of our study was that only a few human cases were collected; more human cases are needed to analyze the correlation of H3K27me3 levels with *HOX* gene expression in human NTDs.

## Conclusions

In conclusion, environmental factors have a profound influence on neural tube closure. Our study showed that epigenetic modifications of H3K27me3 could cause abnormal *Hox* gene expression after exposure to a detrimental environment factor, such as RA, which may significantly contribute to development and etiology of NTDs. The present study provided novel insight into the synergistic function of transcriptional dysregulation and epigenetic modifications. It may be possible to identify novel measures to prevent this devastating birth defect. These results provide evidence supporting the hypothesis that deregulation of *Hox* gene expression through epigenetic mechanisms may be associated with human NTDs. Our findings have broad implications for the mechanisms underlying epigenetic memory which is currently under investigation.

## Methods

### Animals

C57BL/6 mice (44007200007011, 9–10 weeks, 18–23 g) were purchased from Guangdong Medical Laboratory Animal Center (Guangzhou, China), and housed in SPF cage, approved facility on a 12-h light/dark cycle. NTDs mouse embryos were induced by gavage with 28 mg/kg of RA (Sigma, USA, dissolved in sesame oil) on E7.5. On E8.5, E9.5 and E10.5, pregnant mice were euthanized by cervical dislocation and the cranial neural tissue of embryos was collected according to the previous study [37]. All procedures involving animal handling were approved by the Animal Research Ethics Board of Guangdong medical laboratory animal center in China, and were in compliance with institutional guidelines on the care of experimental animals.

### Embryonic stem cell culture and RA treatment

Sv/129 mouse embryonic stem cells (ESCs), were obtained from Xuanwu Hospital (Beijing, China), and maintained in Dulbecco’s modified Eagle’s medium (DMEM, Gibco, USA) supplemented with 0.1 mM β-mercaptoethanol (Invitrogen, Carlsbad, USA), non-essential amino acids (Invitrogen, Carlsbad, USA), 2 mM glutamate (Invitrogen, Carlsbad, USA), 15% fetal bovine serum (Gibco, USA), and 1000 U/ml leukemia inhibitory factor (Millipore, Billerica, USA), cultured in the culture dishes coated with 0.2% gelatin (Invitrogen, Carlsbad, USA). Cells were incubated at 37 °C/5% CO_2_ and passaged every 2 days. ESCs were treated with 1 μM RA for 24 h [38].

### Human samples collection

Normal and NTDs case subjects were obtained from Linxian and Liulin counties, located in the north of Shanxi Province of China. The enrolled pregnant women were diagnosed by trained local clinicians using ultrasonography and then registered in a database. 39 NTDs-affected fetuses and 39 age-matched controls were collected, and the neural tissue samples were used in the following experiments. The sample information was described in Additional file 9: Table S4. The Committee of Medical Ethics in the Capital Institute of Pediatrics (Beijing, China) approved this study (SHERLLM2014002). Written informed consent was obtained from the parents on behalf of the fetuses.

### RNA extraction, cDNA library construction and Illumina sequencing

Total RNA was extracted from mouse embryo cranial neural tissue tissues using the Trizol method (Invitrogen, USA). RNA concentration and quality were assessed by Agilent 2100 Bioanalyzer. The cDNA library was constructed using a TruSeq RNA Sample Preparation Kit (RS-122-2101, Illumina, USA) according to the manufacturer’s protocols. And then, the library could be sequenced using PE91 + 8+91 of Illumina HiSeq™ 2000. After quality of control (QC), the clean reads were obtained and have been deposited into NCBI SRA (accession numbers: SRP070626).

### Bioinformatic analysis of RNA-seq and *Hox* genes screening

The gene expression level is calculated by using RPKM method [39] (reads per kilobase transcriptome per million mapped reads), and the unsupervised clustering analysis was performed using R. The differentially expressed genes (DEGs) were identified by the combination of *P* value < 0.05, FDR < 0.001 and the absolute value of log_2_ ratio > 1, which expression patterns were analyzed by Cluster 3.0 [40] and Java Treeview. The Gene Ontology (GO) and pathway enrichment analysis were used to identify the significantly enriched functional classification, signaling and metabolic pathways of DEGs, which were performed based on Gene Ontology Database (http://www.geneontology.org/) [41] and KEGG pathway database (http://www.genome.jp/kegg/) [42]. GO terms were identified to be significantly enriched when corrected *P* value < 0.05. Pathways were identified to be significantly enriched when FDR < 0.05 meantime. The DEGs were selected by taking intersection among E8.5, E9.5 and E10.5, and the important *Hox* genes were screened by fold change > 20 at E9.5 and E10.5 meantime.

### RT-qPCR

To validate the RNA-seq findings, we prepared new mouse cranial neural tissue of E10.5, and some DEGs were selected and performed with RT-qPCR. In addition, we also validated the important *Hox* genes expression in Sv/129 mouse ESCs treated with 1 μM RA. Total RNA was extracted using the Trizol method (Ambion, USA), first-strand synthesis was done with RevertAid First Strand cDNA Synthesis Kit (Thermo, USA). Maxima SYBR Green/ROX qPCR Master Mix (Thermo, USA) were used for qPCR and the procedure was as follows: (50 °C, 2 min) × 1 cycle; (95 °C, 10 min) × 1 cycle; (95 °C, 15 s; 60 °C, 30 s; 72 °C, 30 s) × 40 cycles; collect fluorescence at 72 °C. Primer sequences were shown in Additional file 10: Table S5.

### Protein extraction

Core histone proteins of cells were extracted using acid extraction [43]. Briefly, the brain tissue was first homogenized in lysis buffer [10 ml solution containing 10 mM Tris–HCl with pH 8.0, 1 mM KCl, 1.5 mM MgCl_2_ and 1 mM dithiothreitol (DTT)] and chilled on ice. 5% of protease inhibitors were added immediately before lysis of cells, chilled on ice for 30 min, and nuclei were isolated by centrifugation (1500*g* for 5 min). For the preparation of histones, nuclei were incubated with four volumes of 0.2 M sulfuric acid (H_2_SO_4_) for overnight at 4 °C. The supernatant was precipitated with 33% trichloroacetic acid (final concentration) and followed by centrifugation (12,000*g* for 5 min at 4 °C). The obtained pellet was washed with cold acetone and subsequently dissolved in distilled water. Core histone proteins of mouse and human brain samples were extracted using EpiQuik™ Total Histone Extraction kit (EPIGENTEK, Farmingdale, NY) according to the manufacturer’s protocols. Nucleoprotein extraction was extracted from cells or mouse and human brain samples using Nucleoprotein Extraction Kit (Sangon Biotech, China) according to the manufacturer’s protocols.

### Western blotting

5 μg of histone was separated on a 12% SDS-PAGE for H3K27me3 and H3 detection. The blots were incubated with the primary antibody, mouse anti- H3K27me3 (1:800, Abcam, Cambridge, UK), anti-H3K27me2, anti-H3K27me1, anti- Suz12 monoclonal antibody (1: 500, Cell Signaling Technology, USA), and mouse anti-H3 monoclonal antibody (1:1,500,000, Abcam, Cambridge, UK) overnight at 4 °C, and then incubated with secondary anti-mouse HRP conjugated antibody (1:5000, Santa, USA) for 1 h at room temperature. The blots were developed with SuperSignal West Pico Chemiluminescence Substrate (Thermo, USA) and quantitated on densitometer (Bio-Rad, Universal HoodII, USA) using Quantity One software.

### Immunofluorescence

Hep-G2 cells were maintained in DMEM (Gibco, USA) supplemented with 10% fetal bovine serum (Gibco, USA), treated with 1 μM RA for 24 h. The primary antibodies used for immunofluorescence staining were the mouse monoclonal anti-H3K27me3 antibody (1: 200, Abcam, UK).

### Immunohistochemistry

Embryos (E10.5) were fixed in 4% paraformaldehyde overnight and processed to generate 5-μm paraffin sections. Immunohistochemistry was performed on transverse sections according to the method previously described [44]. The primary antibodies used were the mouse monoclonal anti-H3K27me3 antibody (1: 200, Abcam, UK). The area percentage of the H3K27me3 positive was analyzed by Image J software.

### ChIP-Seq and data analysis

Cranial neural tissue of pooled mouse embryos on E10.5 were collected and performed by ChIP-seq. ChIP was done with Simple ChIP Enzymatic chromatinIP kit (9003s) from Cell Signaling Technology and chromatin was sheared to an average DNA fragment size of 100–300 bp. ChIP-Seq libraries were prepared according to Illumina protocols. Sequencing was done with Illumina HiSeq platform. After quality of control (QC), the clean reads were obtained and have been deposited into NCBI SRA (Accession Numbers: SRP193168). The clean data were mapped to the mouse genome (mm9) using SOAPaligner/SOAP2 (Version: 2.21t), and no more than two mismatches are allowed in the alignment. Peaks of H3K27me3 ChIP-Seq signals on genome were determined using MACS2 with false-discovery rate as 0.05. Tracks of H3K27me3 ChIP-seq were viewed by UCSC Genome Browser for mm9.

### Chromatin immunoprecipitation (ChIP) analysis

ChIP assays were performed using the SimpleChIP Enzymatic chromatin IP system (Cell Signaling, California, USA) following the manufacturer’s protocols. Chromatin was prepared, sonicated to DNA segments between 200 and 1000 bp and then immunoprecipitated with anti-H3K27me3, anti-UTX (Abcam, Cambridge, UK) and anti-H3K27me2 (Cell Signaling Technology, USA). The immunoprecipitated DNA was analyzed by qPCR, which were performed using QuantStudio 7 Flex with SYBR Green detection. The primers used for ChIP assays were shown in Additional file 11: Table S6 [45–53]. Mouse IgG antibodies were used as negative controls in the immunoprecipitations. The following equation was used to calculate percent input = 2% × 2^(CT) 2% input sample − (CT) IP sample)^.

### Human mRNA detection

NanoString nCounter system was used to analyze the 10 *HOX* mRNA expression level of degradative brain tissues from human fetus samples. The RNA was extracted using miRNeasy Mini Kit (Qiagen, Germany). Hybridizations were performed according to the NanoString miRGE Assay Manual. Approximately 100 ng of each RNA sample was mixed with 20 μl of nCounter Reporter probes in hybridization buffer and 5 μl of nCounter Capture probes for a total reaction volume of 30 μl. The hybridizations were incubated at 65 °C for approximately 16 h, then eluted and immobilized in the cartridge for data collection, which was performed on the nCounter Digital Analyzer. The counts were analyzed by log_2_ transformation using nSolver Analysis Software 2.5, and normalized by housekeeping genes such as GAPDH, CLCT, GUSB, HPRT1 and PKG1 genes.

### UTX activity detection

UTX activity was detected according to the manufacturer’s protocols of Epigenase JMJD3/UTX Demethylase activity/inhibition assay kit (Epigentek, Farmingdale, NY) using 10 μg of nuclear extracts. The UTX activity was calculated as the following formula: UTX activity (OD/min/mg) = sample OD − Blank OD/(protein amount (μg) × min) × 1000.

### UTX inhibitor

Sv/129 ESCs were treated with 30 nM of UTX inhibitor GSK J4 sc-391114 (Santa Cruz, USA) for 6 h according to the manufacturer’s protocol.

### Suz12 siRNA and overexpression of transfection

Mouse F9 cells were maintained in DMEM (Gibco, USA) supplemented with 10% fetal bovine serum (Gibco, USA). siRNA was delivered to cells using Lipofectamine 2000 according to the manufacturer’s instruction. siRNAs specific for Suz12 5′-AAGCTGTTACCAAGCTCCGTG-3′ and a nonspecific siRNA 5′-TTCTCCGAACGTGTCACGT-3′ were designed and synthesized (Sangon Biotech, China), and the latter was transfected as negative control. Suz12 expression plasmid was purchase from Origene. After transfection for 24 h, F9 cells were treated with 1 μM RA for 24 h, and then harvested for further analysis.

### Statistical analysis

Statistical analysis was performed using SPSS software, version 22.0 (SPSS, Inc., Chicago, IL, USA). Data were expressed as the mean ± SD, Student’s *t* test or ANOVA analysis was performed. And Pearson’s correlation analysis was used to analyze the association between *HOX*s expression and H3K27me3 level*. P *< 0.05 was considered statistically significant.

### Ethics statement

All animals were handled in strict accordance with the “Guide for the Care and Use of Laboratory Animals” and the “Principles for the Utilization and Care of Vertebrate Animals”, and all animal work was approved by Institutional Animal Care and Use Committee (IACUC) at the Beijing Institute of Radiation Medicine. The study using clinical samples including 39 paired human NTDs and matched normal tissues were approved by department of Lvliang area of Shanxi Province in northern China. Informed consent was obtained from all subjects or their relatives. Human samples were collected and analyzed in accordance with Capital Institute of Pediatrics approval. The Ethics Board of Capital Institute of Pediatrics approved the study protocol. All animal experiments were conducted in compliance with the guidelines of the Institute for Laboratory Animal Research, Capital Institute of Pediatrics.

## Supplementary information

**Additional file 1: Figure S1.** Bioinformatic analysis of RNA-seq. **A.** Morphology of mouse NTDs embryos induced by RA. (**a).** Normal mouse embryo. (**b).** Mouse embryo showed growth retardation, neural tube close incompletely. Arrow indicates unclosed neural tube. (**c).** Mouse embryo showed anencephaly, enlarged heart and ventricular chambers, and short tail. Arrow indicates hindbrain, heart and tail respectively. **B.** Unsupervised hierarchical clustering plot of genes detected in mouse embryo cranial neural tissue. **C.** GO functional classification of DEGs. Blue represents cellular component, red represents molecular function, and green represents biological process. **D.** KEGG pathway analysis of DEGs. **E.** Protein-protein interaction (PPI) network of 196 genes analyzed by STRING database.

**Additional file 2: Table S1.** Enriched GO terms of DEGs in Con-E8.5-vs-RA-E8.5, Con-E9.5-vs-RA-E9.5 and Con-E10.5-vs-RA-E10.5 comparisons.

**Additional file 3: Table S2.** Enriched KEGG pathways of DEGs in Con-E8.5-vs-RA-E8.5, Con-E9.5-vs-RA-E9.5 and Con-E10.5-vs-RA-E10.5 comparisons.

**Additional file 4: Figure S2.** H3K27me3/2/1 analysis in mouse NTD embryos of E10.5. **A.** Relative protein expression of H3K27me3, H3K27me2 and H3K27me1 in mouse NTDs embryo. Data are shown as the mean (SD; n= 3). *P < 0.05. **B.** Peak statistics of mouse embryos used for ChIP-seq. **C.** GO analysis of differential peak related gene.

**Additional file 5: Table S3.** Differential peaks related genes of mouse NTDs embryos of E10.5.

**Additional file 6: Figure S3.** H3K27me3/2/1 analysis in RA-induced ESCs. **A.** Relative protein expression of H3K27me3, H3K27me2 and H3K27me1in RA-induced ESCs. Data are shown as the mean (SD; n= 3). *P < 0.05. **B.** ChIP assays of H3K27me2 were performed using F9 cells treated with 1 μM RA for 24 h. Mouse IgG was used as control. Enrichment of Hox gene promoters was measured by qPCR.

**Additional file 7: Figure S4.** Suz12 and Ezh2 decreased in RA-induced mouse NTDs and ESCs. **A.** RNA-seq analysis showed Suz12 expression in cranial neural tissue of RA-induced mouse NTDs embryos from E8.5 to E10.5. **B.** RNA-seq analysis showed Ezh2 expression in cranial neural tissue of RA-induced mouse NTDs embryos from E8.5 to E10.5. **C.** Ezh2 level in cranial neural tissue of RA-induced mouse NTDs was measured by RT-qPCR and Western blotting. *Actb* and Gapdh were used as a loading control respectively. Data are shown as the mean (SD; n= 4). *P < 0.05. **D.** Ezh2 level in mouse ESCs treated with RA was measured by RT-qPCR and Western blotting. *Actb* and Gapdh were used as loading control respectively. Data are shown as the mean (SD; n= 3). *P < 0.05. **E.** Relative protein expression of H3K27me3 after overexpression of Suz12 in RA-induced F9 cells. Data are shown as the mean (SD; n= 3). Different letters represent the difference had statistic significance, *P* < 0.05.

**Additional file 8: Figure S5.** *HOX* gene expression in human spinal bifida, hydrocephaly and encephalocele. **A.** *HOX* genes were detected with NanoString in the spinal cord from human spinal bifida and normal cases. Data are shown as the mean (SD; n= 10). *P* < 0.05 indicates statistical significance. **B.** *HOX* genes were detected with NanoString in the brain tissues from human hydrocephaly and normal cases. Data are shown as the mean (SD; n= 10). *P* < 0.05 indicates statistical significance. **C.** *HOX* genes were detected with NanoString in the brain tissues from human encephalocele and normal cases. Data are shown as the mean (SD; n= 9). *P* < 0.05 indicates statistical significance. **D.** Whole genome sequencing of 100 human NTDs samples. **E, F.** Variant distribution allele frequency of 10 *HOX* genes in 100 human NTDs samples. **G.** The number of variants by minor allele frequency (MAF) in 100 human NTDs samples. **H, I.** Variant rate of 10 *HOX* genes in 100 human NTDs samples.

**Additional file 9: Table S4.** Information of human NTDs samples. The human cases with anencephaly, spinal bifida, hydrocephaly and encephalocele involved in this study. Gender, gestational age, sample type and NTDs phenotype are listed.

**Additional file 10: Table S5.** RT-qPCR primer sequences. All oligonucleotides were synthesized by Sangon Biotech.

**Additional file 11: Table S6.** ChIP-qPCR primer sequences. All oligonucleotides were synthesized by Sangon Biotech.

## Data Availability

All the data generated or analyzed during this study are included in this article and its supplementary information files.

## Abbreviations

ChIP: chromatin immunoprecipitation
CNS: central nervous system
DEGs: differentially expressed genes
DMEM: Dulbecco’s modified Eagle’s medium
ESCs: embryonic stem cells
GO: Gene Ontology
*Hox*: Homeobox Gene
MAF: minor allele frequency
NTDs: neural tube defects
PRC2: polycomb repressive complexe 2
PPI: protein–protein interaction
QC: quality of control
RA: retinoic acid
RNA-seq: RNA sequencing
RPKM: reads per kilobase transcriptome per million mapped reads
