# Supplementary figures and images for: Reduced H3K27me3 leads to abnormal Hox gene expression in neural tube defects
Source: Epigenetics Chromatin. 2019 Dec 19;12:76. doi: 10.1186/s13072-019-0318-1 (PMC6921514; doi:10.1186/s13072-019-0318-1)

# Figure S1

**A**

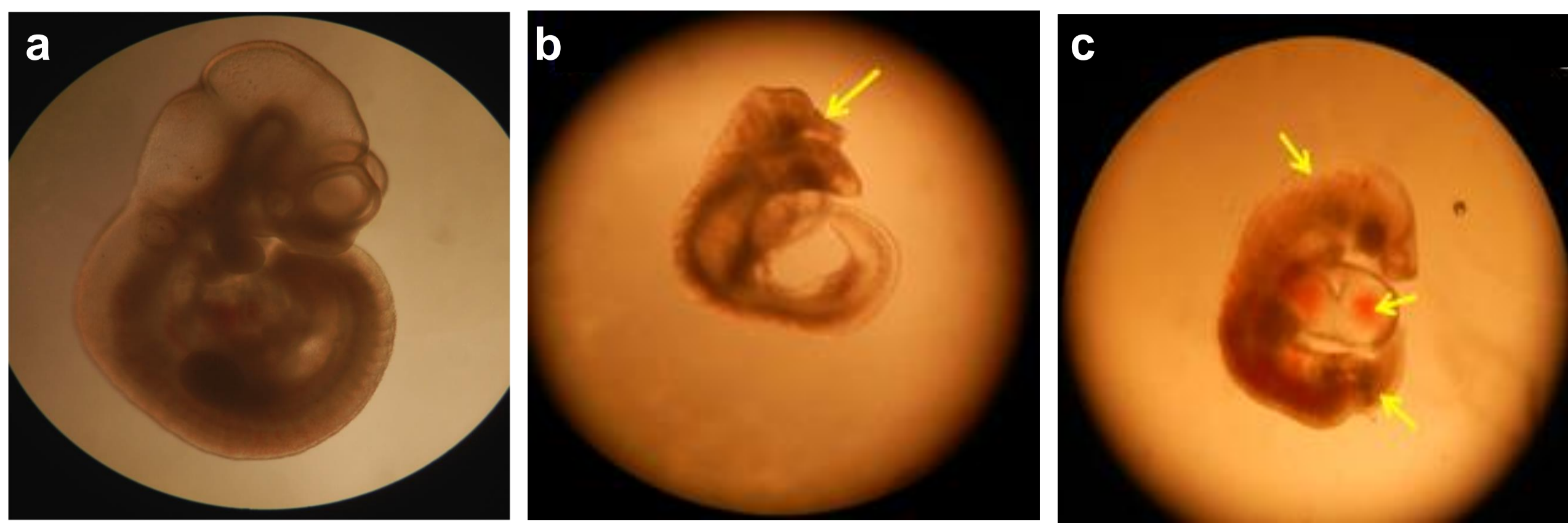

# B

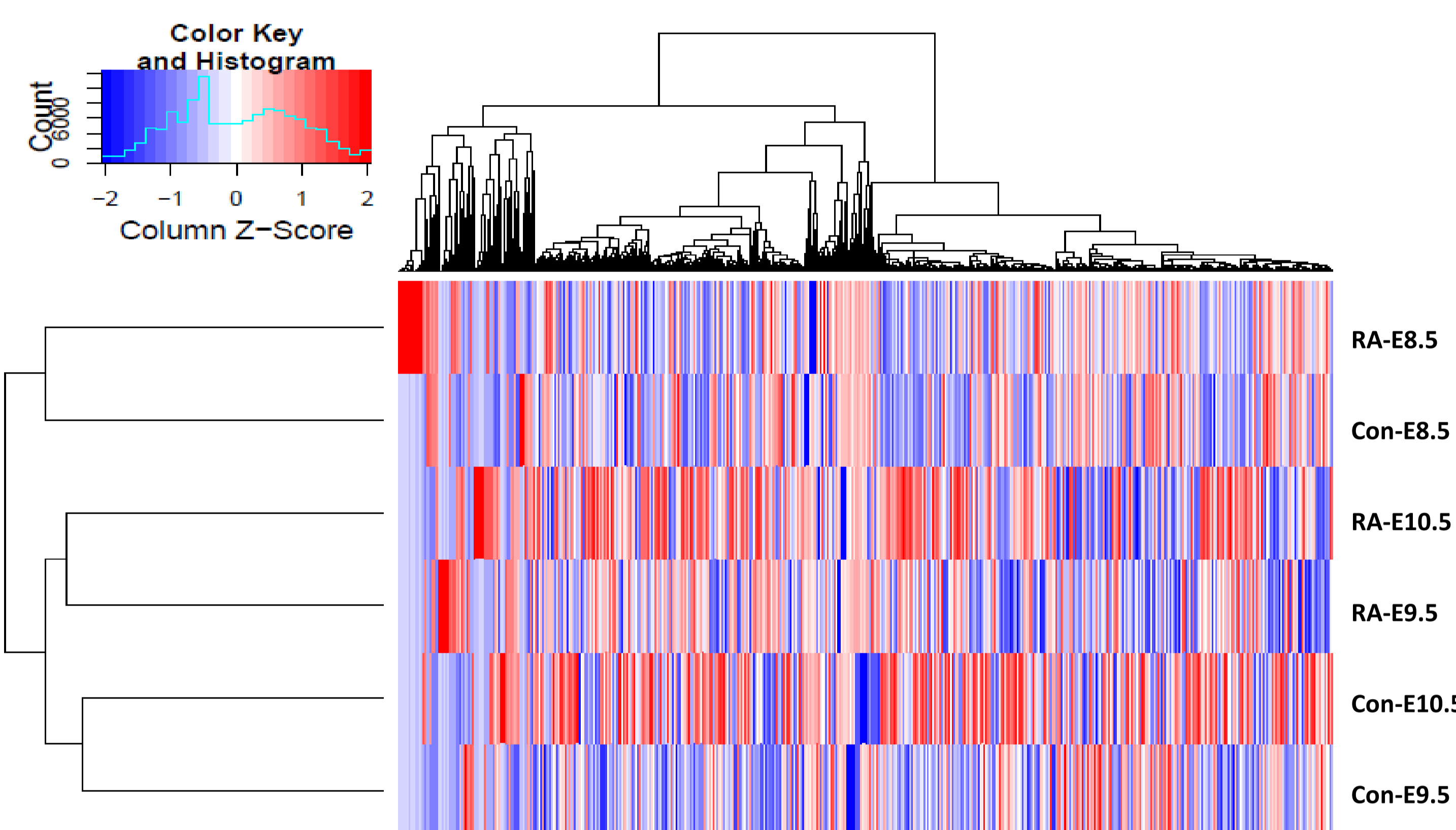

# D

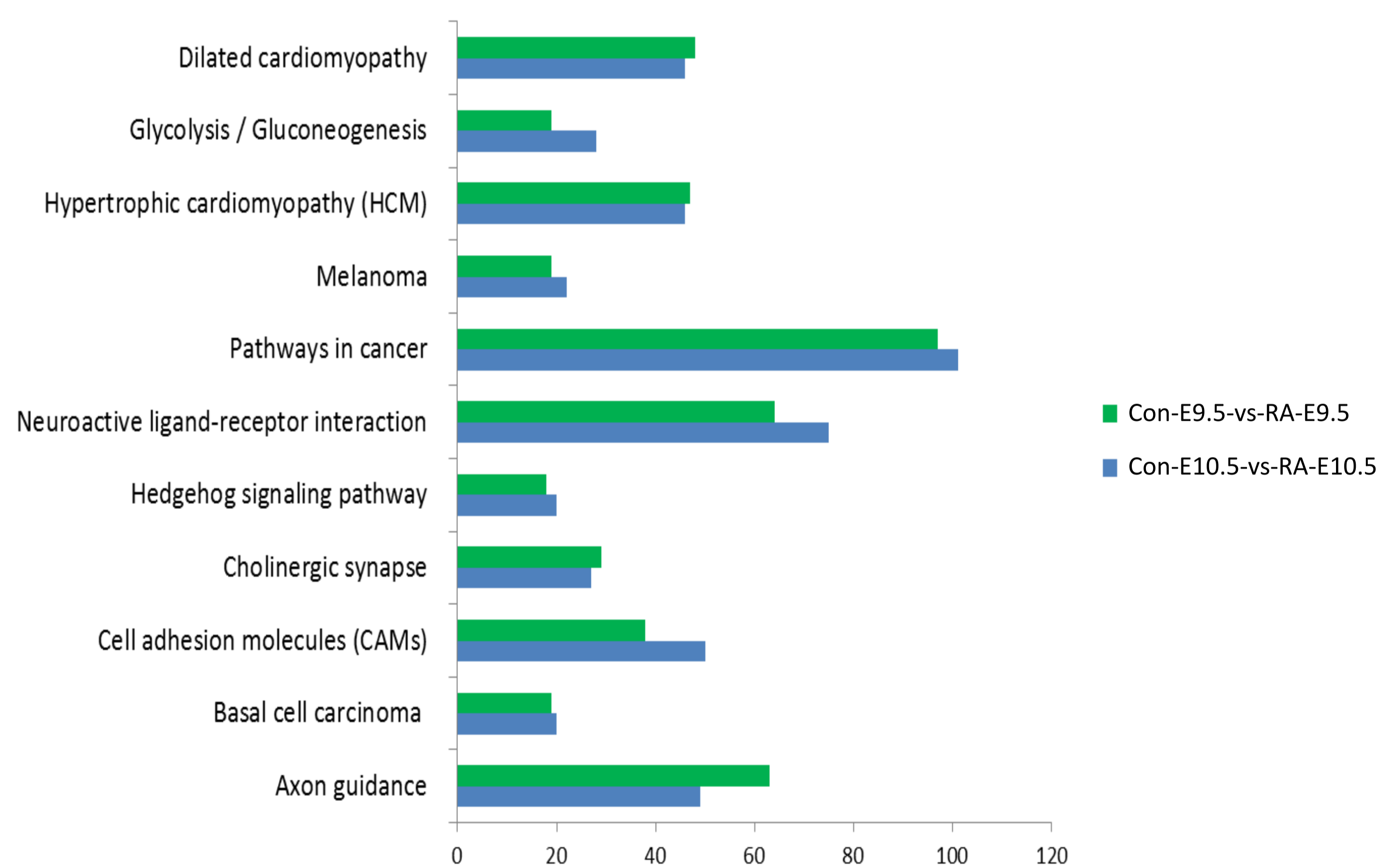

C

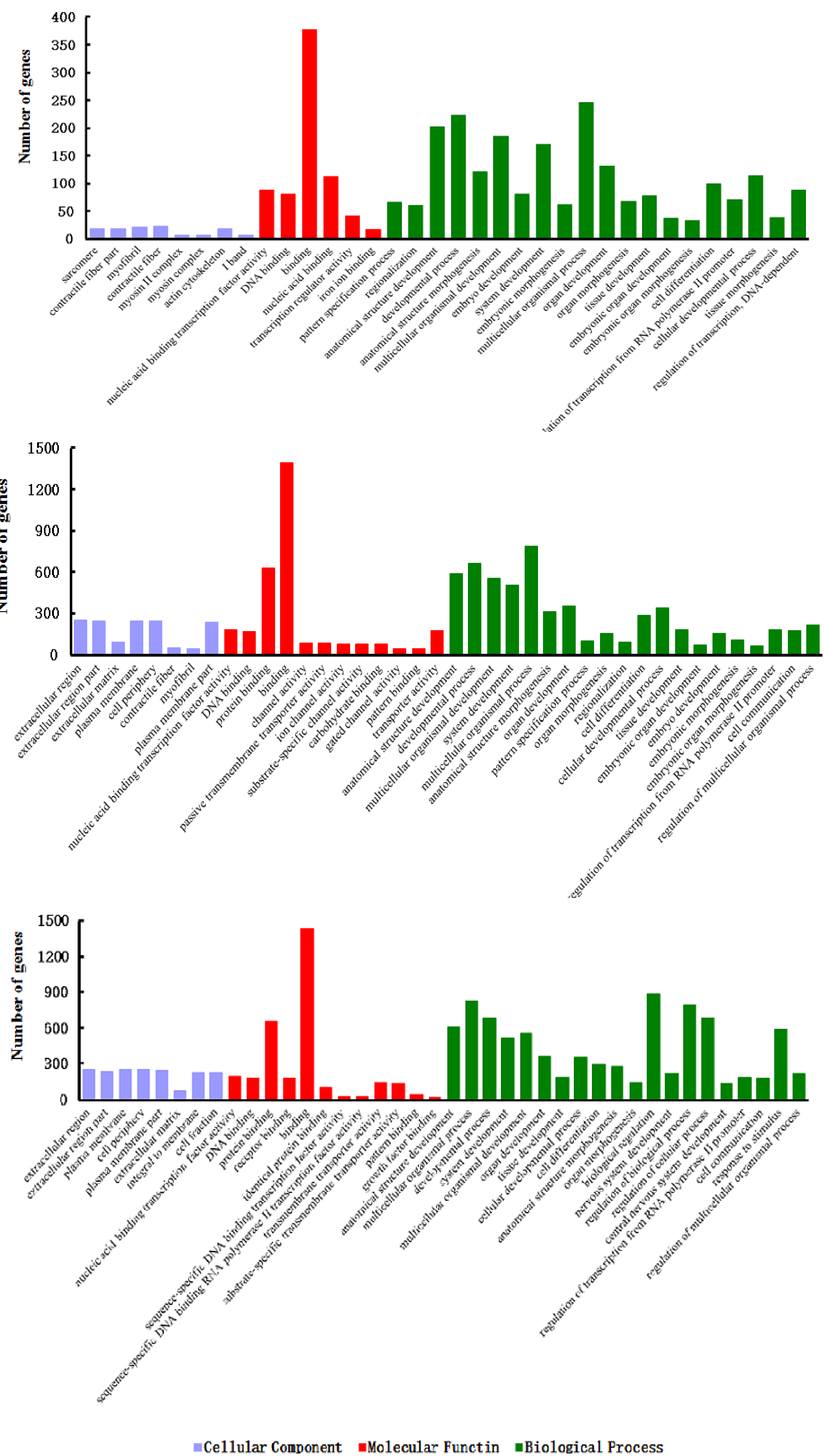

# E

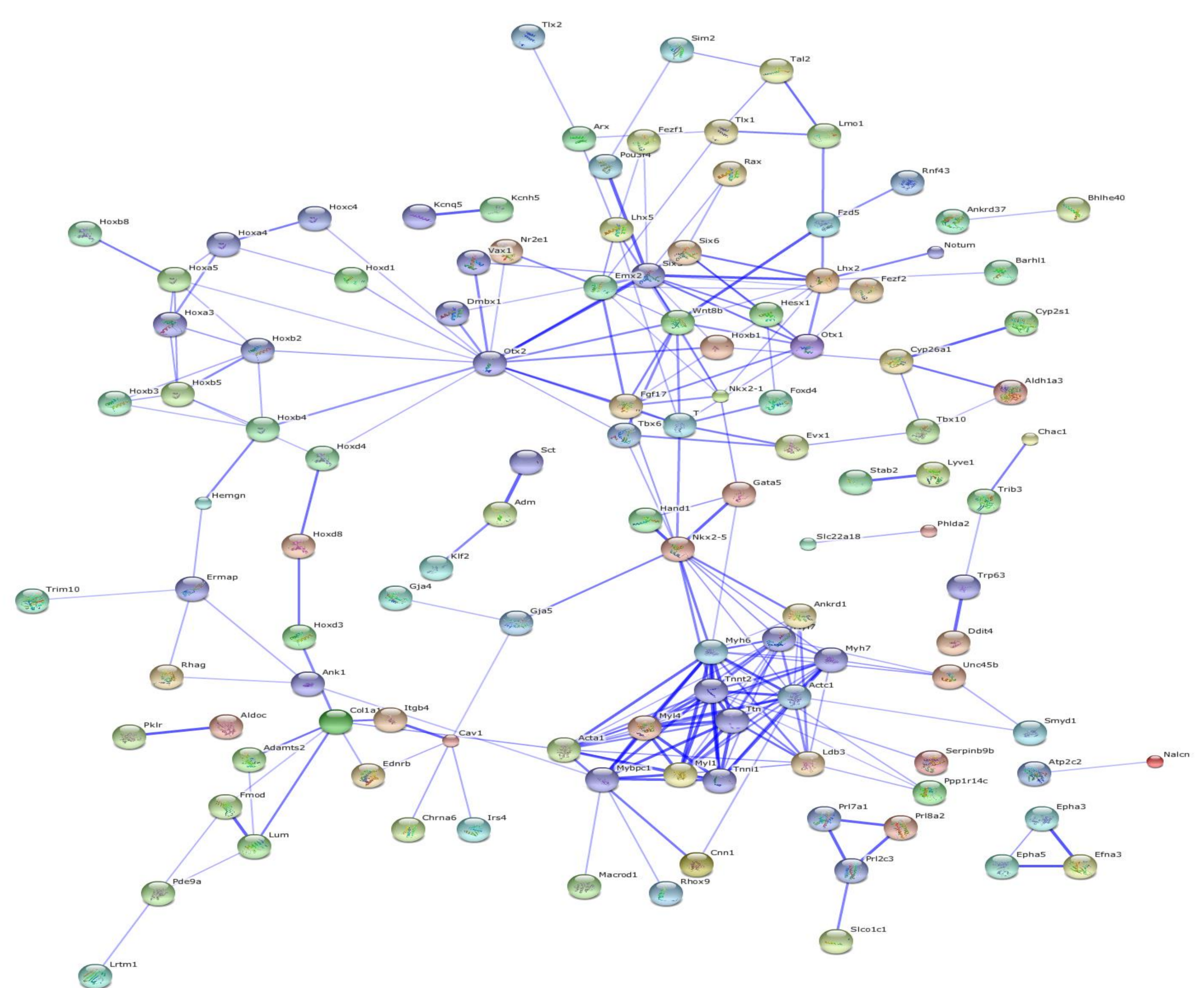

Supplement: Supplementary file 1 — Additional file 1: Figure S1. Bioinformatic analysis of RNA-seq. A. Morphology of mouse NTDs embryos induced by RA. (a). Normal mouse embryo. (b). Mouse embryo showed growth retardation, neural tube close incompletely. Arrow indicates unclosed neural tube. (c). Mouse embryo showed anencephaly, enlarged heart and ventricular chambers, and short tail. Arrow indicates hindbrain, heart and tail respectively. B. Unsupervised hierarchical clustering plot of genes detected in mouse embryo cranial neural tissue. C. GO functional classification of DEGs. Blue represents cellular component, red represents molecular function, and green represents biological process. D. KEGG pathway analysis of DEGs. E. Protein-protein interaction (PPI) network of 196 genes analyzed by STRING database. [file 13072_2019_318_MOESM1_ESM.pdf]

Figure S3

A

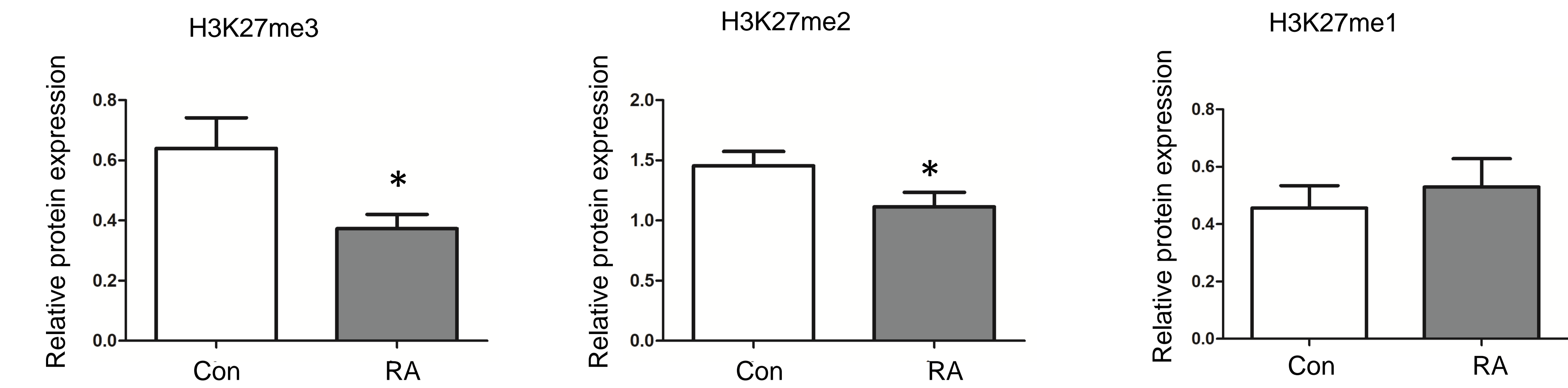

B

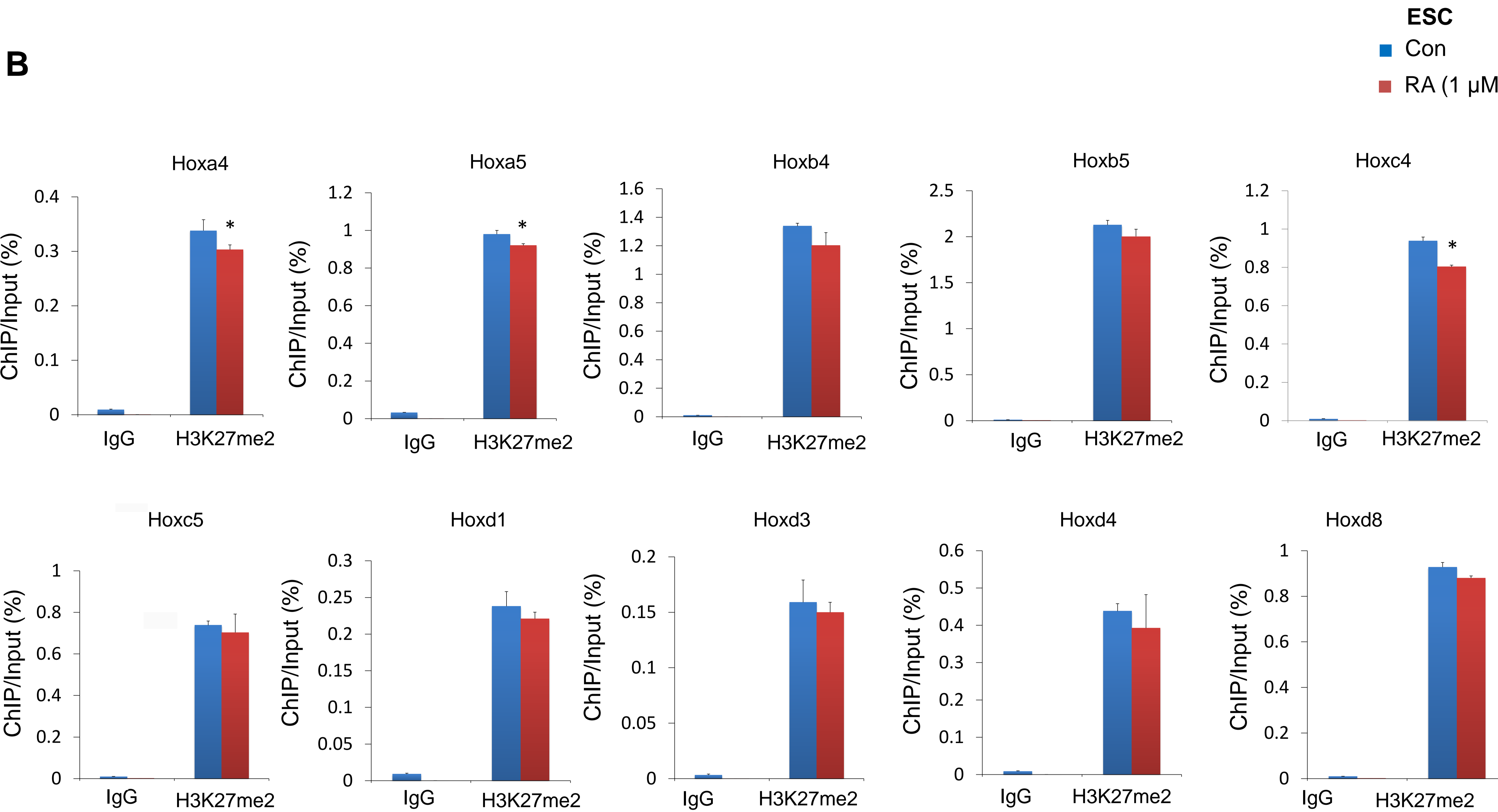

Supplement: Supplementary file 6 — Additional file 6: Figure S3. H3K27me3/2/1 analysis in RA-induced ESCs. A. Relative protein expression of H3K27me3, H3K27me2 and H3K27me1in RA-induced ESCs. Data are shown as the mean (SD; n= 3). *P < 0.05. B. ChIP assays of H3K27me2 were performed using F9 cells treated with 1 μM RA for 24 h. Mouse IgG was used as control. Enrichment of Hox gene promoters was measured by qPCR. [file 13072_2019_318_MOESM6_ESM.pdf]

Figure S4

A

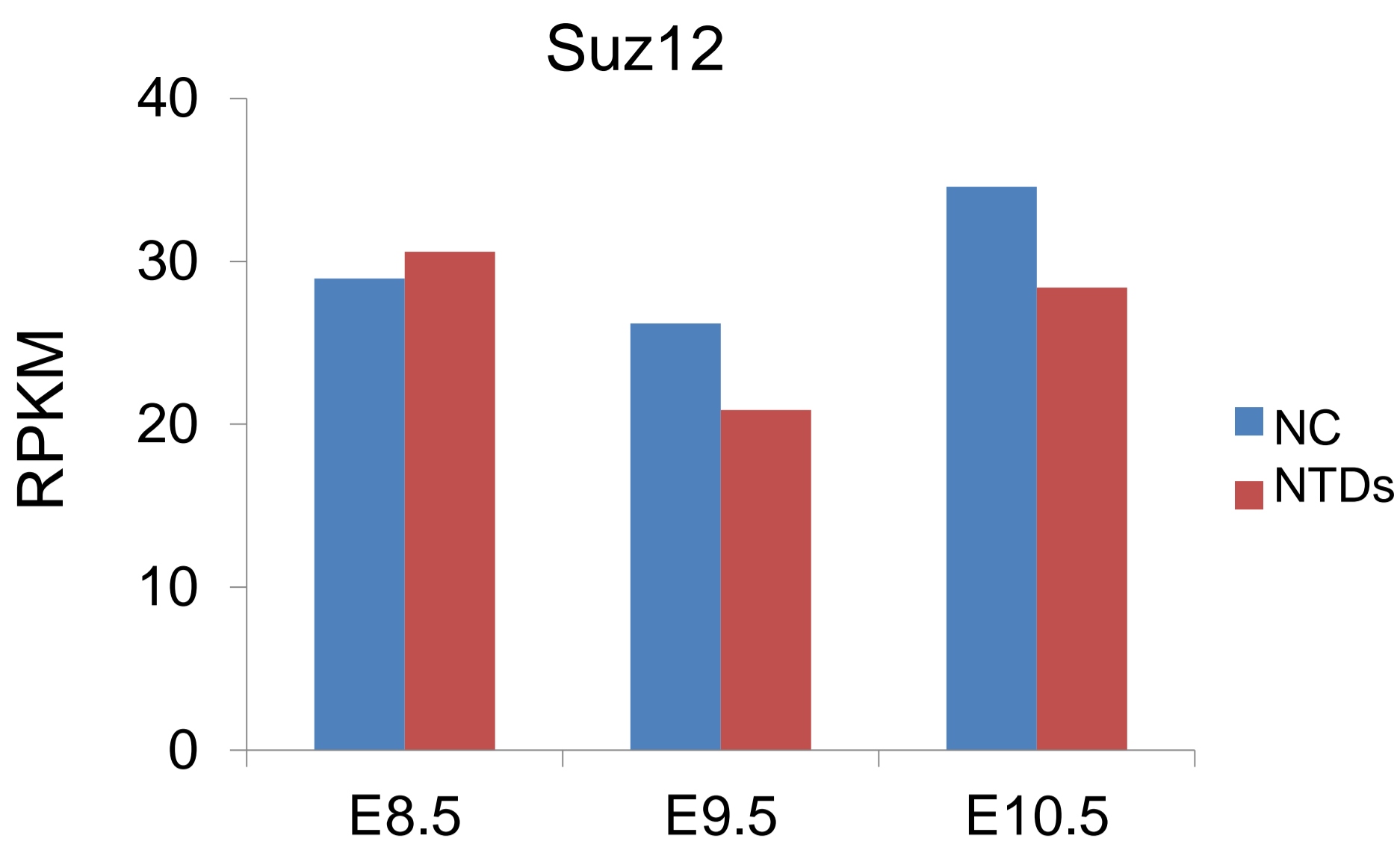

B

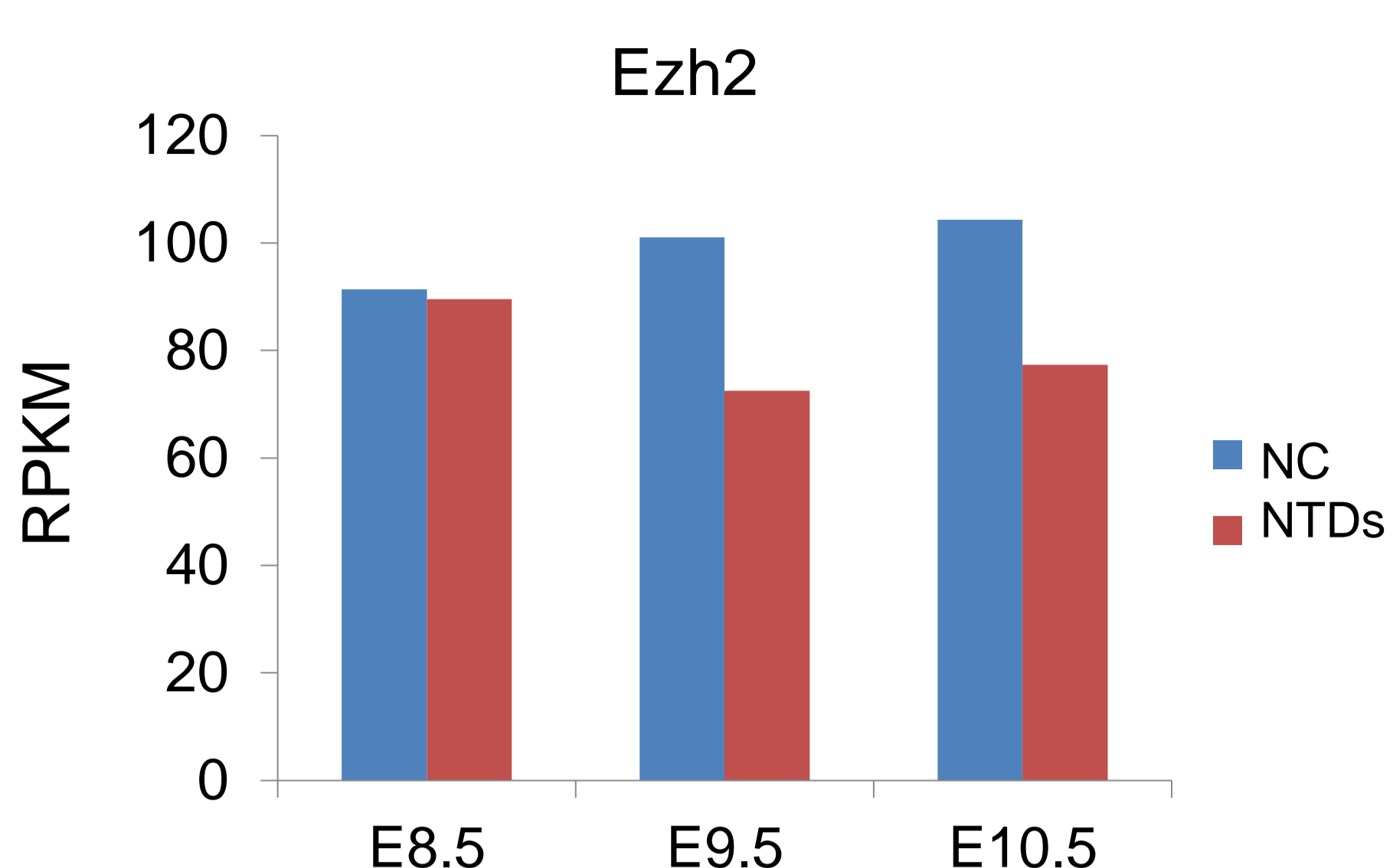

C

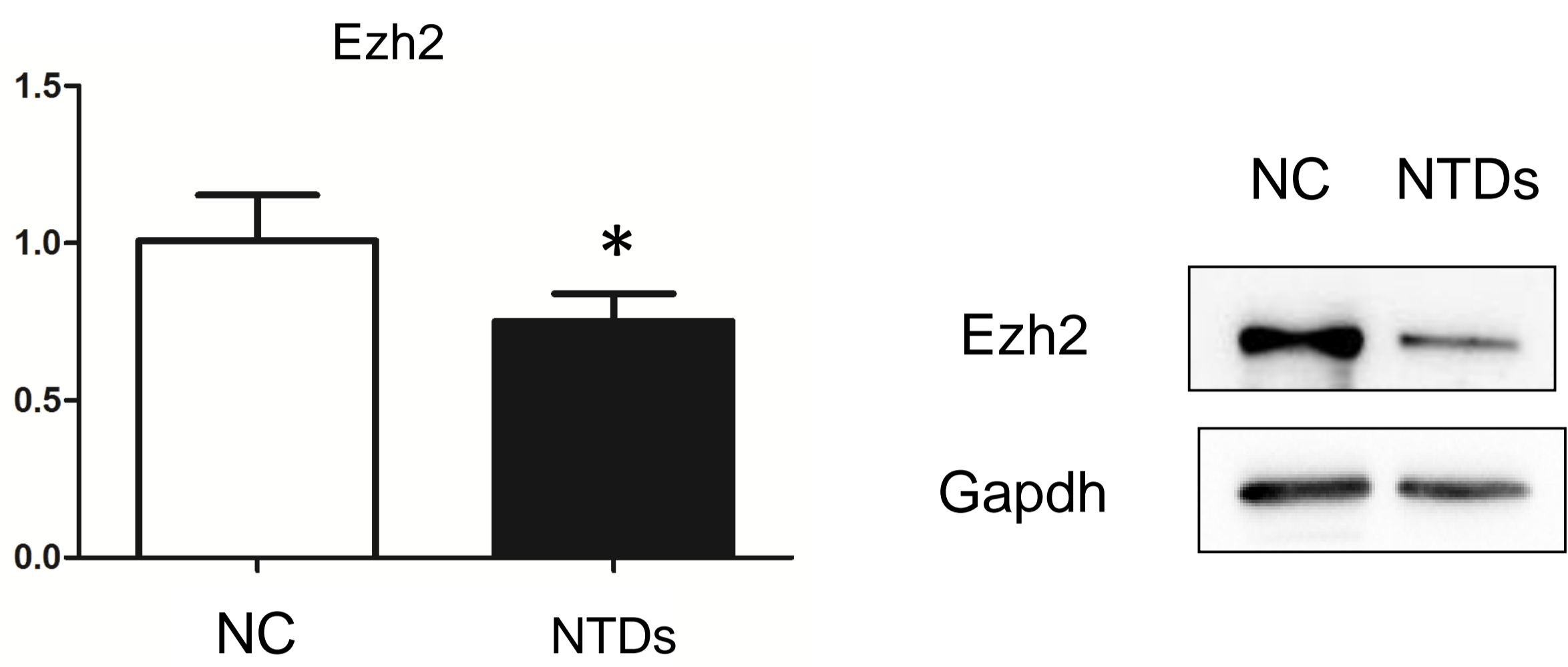

D

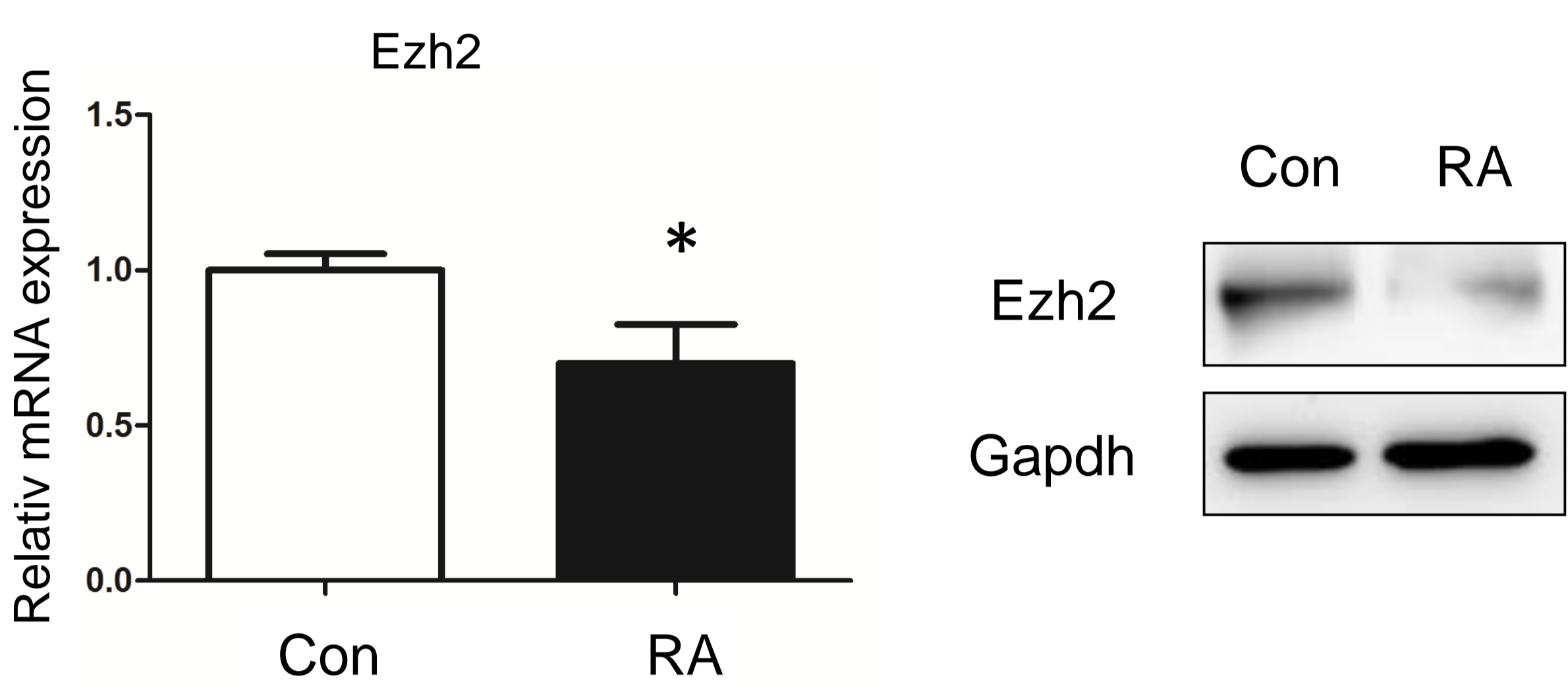

E

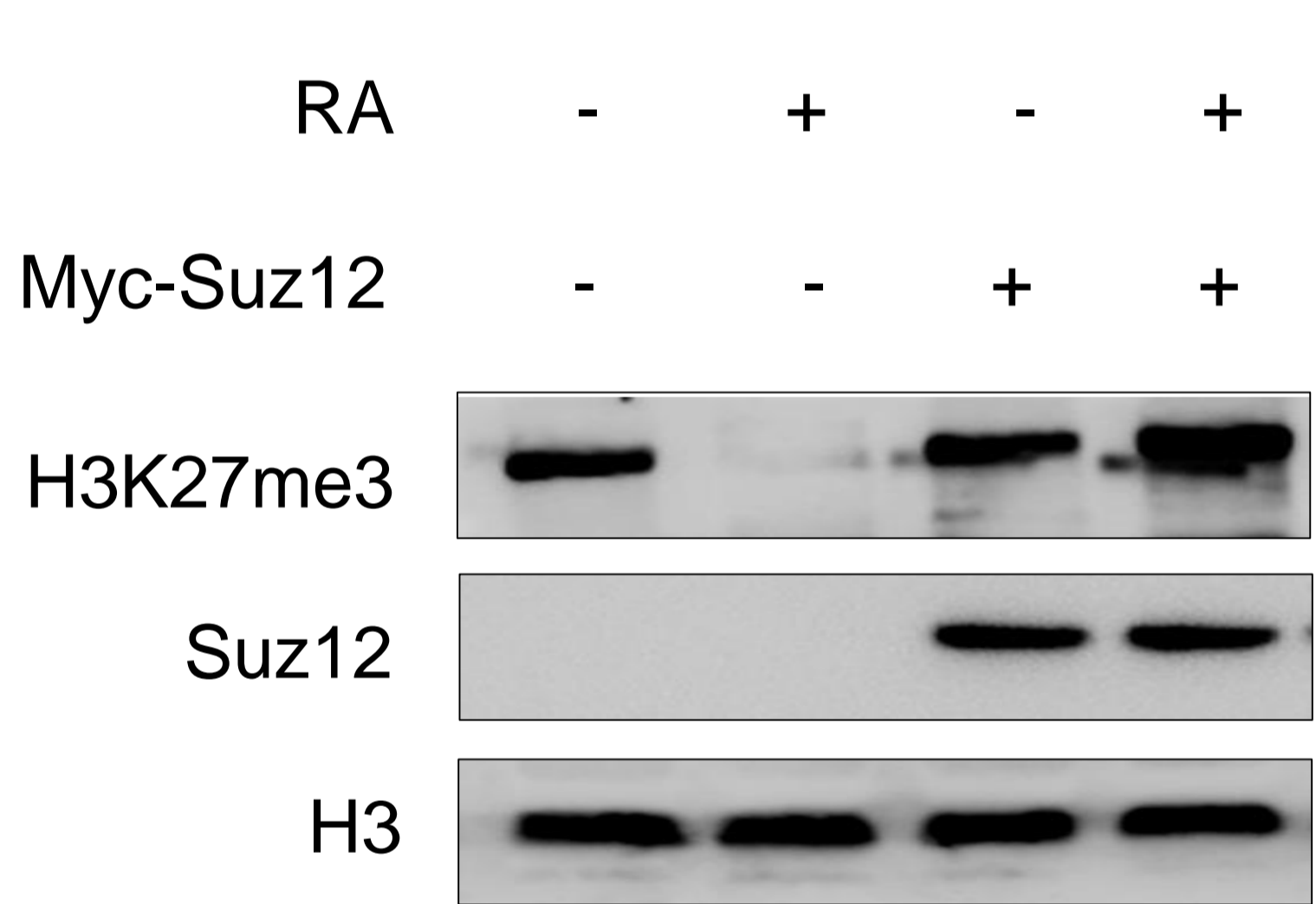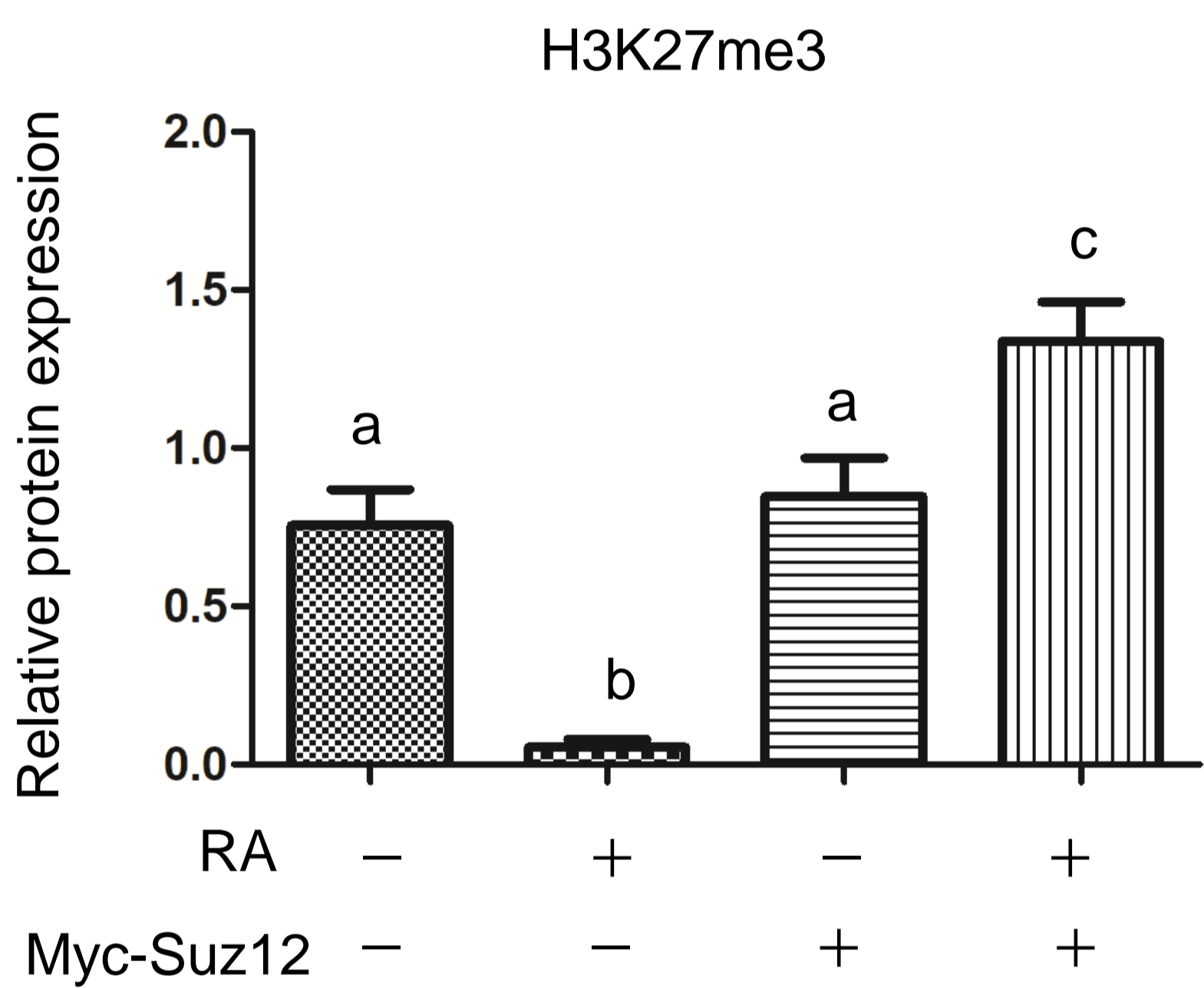

Supplement: Supplementary file 7 — Additional file 7: Figure S4. Suz12 and Ezh2 decreased in RA-induced mouse NTDs and ESCs. A. RNA-seq analysis showed Suz12 expression in cranial neural tissue of RA-induced mouse NTDs embryos from E8.5 to E10.5. B. RNA-seq analysis showed Ezh2 expression in cranial neural tissue of RA-induced mouse NTDs embryos from E8.5 to E10.5. C. Ezh2 level in cranial neural tissue of RA-induced mouse NTDs was measured by RT-qPCR and Western blotting. Actb and Gapdh were used as a loading control respectively. Data are shown as the mean (SD; n= 4). *P < 0.05. D. Ezh2 level in mouse ESCs treated with RA was measured by RT-qPCR and Western blotting. Actb and Gapdh were used as loading control respectively. Data are shown as the mean (SD; n= 3). *P < 0.05. E. Relative protein expression of H3K27me3 after overexpression of Suz12 in RA-induced F9 cells. Data are shown as the mean (SD; n= 3). Different letters represent the difference had statistic significance, P < 0.05. [file 13072_2019_318_MOESM7_ESM.pdf]
