# Supplementary material for: Reduced H3K27me3 leads to abnormal Hox gene expression in neural tube defects
Source: Epigenetics Chromatin. 2019 Dec 19;12:76. doi: 10.1186/s13072-019-0318-1 (PMC6921514; doi:10.1186/s13072-019-0318-1)
Supplement: Supplementary file 2 — Additional file 2: Table S1. Enriched GO terms of DEGs in Con-E8.5-vs-RA-E8.5, Con-E9.5-vs-RA-E9.5 and Con-E10.5-vs-RA-E10.5 comparisons. [file 13072_2019_318_MOESM2_ESM.doc]

**Table S1. Gene Ontology (GO) functional classification of differentially expressed genes (DEGs)**

| Category | Con-E8.5 vs RA-E8.5 | | | |  | Con-E9.5 vs RA-E9.5 | | | |  | Con-E10.5 vs RA-E10.5 | | | |
| --- | --- | --- | --- | --- | --- | --- | --- | --- | --- | --- | --- | --- | --- | --- |
|  | Term | Cluster frequency | Genome frequency of use | Corrected P-value |  | Term | Cluster frequency | Genome frequency of use | Corrected P-value |  | Term | Cluster frequency | Genome frequency of use | Corrected P-value |
| Celluar component | [sarcomere](http://amigo.geneontology.org/amigo/term/GO:0030017) | 19 out of 445 genes, 4.3% | 87 out of 15584 genes, 0.6% | 5.22e-10 |  | [extracellular region](http://amigo.geneontology.org/amigo/term/GO:0005576) | 257 out of 1747 genes, 14.7% | 1184 out of 15584 genes, 7.6% | 4.01e-25 |  | [extracellular region](http://amigo.geneontology.org/amigo/term/GO:0005576) | 255 out of 1803 genes, 14.1% | 1184 out of 15584 genes, 7.6% | 2.76e-22 |
|  | [contractile fiber part](http://amigo.geneontology.org/amigo/term/GO:0044449) | 19 out of 445 genes, 4.3% | 88 out of 15584 genes, 0.6% | 6.49e-10 |  | [extracellular region part](http://amigo.geneontology.org/amigo/term/GO:0044421) | 247 out of 1747 genes, 14.1% | 1146 out of 15584 genes, 7.4% | 1.49e-23 |  | [extracellular region part](http://amigo.geneontology.org/amigo/term/GO:0044421) | 242 out of 1803 genes, 13.4% | 1146 out of 15584 genes, 7.4% | 8.85e-20 |
|  | [myofibril](http://amigo.geneontology.org/amigo/term/GO:0030016) | 22 out of 445 genes, 4.9% | 123 out of 15584 genes, 0.8% | 7.31e-10 |  | [extracellular matrix](http://amigo.geneontology.org/amigo/term/GO:0031012) | 94 out of 1747 genes, 5.4% | 308 out of 15584 genes, 2.0% | 4.21e-18 |  | [plasma membrane](http://amigo.geneontology.org/amigo/term/GO:0005886) | 260 out of 1803 genes, 14.4% | 1391 out of 15584 genes, 8.9% | 5.67e-14 |
|  | [contractile fiber](http://amigo.geneontology.org/amigo/term/GO:0043292) | 23 out of 445 genes, 5.2% | 144 out of 15584 genes, 0.9% | 2.71e-09 |  | [plasma membrane](http://amigo.geneontology.org/amigo/term/GO:0005886) | 249 out of 1747 genes, 14.3% | 1391 out of 15584 genes, 8.9% | 1.22e-12 |  | [cell periphery](http://amigo.geneontology.org/amigo/term/GO:0071944) | 260 out of 1803 genes, 14.4% | 1405 out of 15584 genes, 9.0% | 2.04e-13 |
|  | [myosin II complex](http://amigo.geneontology.org/amigo/term/GO:0016460) | 7 out of 445 genes, 1.6% | 16 out of 15584 genes, 0.1% | 1.94e-05 |  | [cell periphery](http://amigo.geneontology.org/amigo/term/GO:0071944) | 250 out of 1747 genes, 14.3% | 1405 out of 15584 genes, 9.0% | 2.20e-12 |  | [plasma membrane part](http://amigo.geneontology.org/amigo/term/GO:0044459) | 249 out of 1803 genes, 13.8% | 1335 out of 15584 genes, 8.6% | 3.58e-13 |
|  | [myosin complex](http://amigo.geneontology.org/amigo/term/GO:0016459) | 8 out of 445 genes, 1.8% | 31 out of 15584 genes, 0.2% | 0.00026 |  | [contractile fiber](http://amigo.geneontology.org/amigo/term/GO:0043292) | 51 out of 1747 genes, 2.9% | 144 out of 15584 genes, 0.9% | 3.53e-12 |  | [extracellular matrix](http://amigo.geneontology.org/amigo/term/GO:0031012) | 84 out of 1803 genes, 4.7% | 308 out of 15584 genes, 2.0% | 4.75e-12 |
|  | [actin cytoskeleton](http://amigo.geneontology.org/amigo/term/GO:0015629) | 19 out of 445 genes, 4.3% | 203 out of 15584 genes, 1.3% | 0.00085 |  | [myofibril](http://amigo.geneontology.org/amigo/term/GO:0030016) | 46 out of 1747 genes, 2.6% | 123 out of 15584 genes, 0.8% | 6.80e-12 |  | [integral to membrane](http://amigo.geneontology.org/amigo/term/GO:0016021) | 233 out of 1803 genes, 12.9% | 1361 out of 15584 genes, 8.7% | 3.10e-08 |
|  | [I band](http://amigo.geneontology.org/amigo/term/GO:0031674) | 8 out of 445 genes, 1.8% | 55 out of 15584 genes, 0.4% | 0.02241 |  | [plasma membrane part](http://amigo.geneontology.org/amigo/term/GO:0044459) | 237 out of 1747 genes, 13.6% | 1335 out of 15584 genes, 8.6% | 1.62e-11 |  | [cell fraction](http://amigo.geneontology.org/amigo/term/GO:0000267) | 236 out of 1803 genes, 13.1% | 1526 out of 15584 genes, 9.8% | 0.00019 |
| Molecular function | [nucleic acid binding transcription factor activity](http://amigo.geneontology.org/amigo/term/GO:0001071) | 88 out of 436 genes, 20.2% | 827 out of 15451 genes, 5.4% | 8.19e-26 |  | [nucleic acid binding transcription factor activity](http://amigo.geneontology.org/amigo/term/GO:0001071) | 187 out of 1674 genes, 11.2% | 827 out of 15451 genes, 5.4% | 3.58e-21 |  | [nucleic acid binding transcription factor activity](http://amigo.geneontology.org/amigo/term/GO:0001071) | 195 out of 1731 genes, 11.3% | 827 out of 15451 genes, 5.4% | 9.56e-23 |
|  | [DNA binding](http://amigo.geneontology.org/amigo/term/GO:0003677) | 81 out of 436 genes, 18.6% | 821 out of 15451 genes, 5.3% | 2.42e-21 |  | [DNA binding](http://amigo.geneontology.org/amigo/term/GO:0003677) | 174 out of 1674 genes, 10.4% | 821 out of 15451 genes, 5.3% | 2.10e-16 |  | [DNA binding](http://amigo.geneontology.org/amigo/term/GO:0003677) | 178 out of 1731 genes, 10.3% | 821 out of 15451 genes, 5.3% | 2.22e-16 |
|  | [binding](http://amigo.geneontology.org/amigo/term/GO:0005488) | 379 out of 436 genes, 86.9% | 11911 out of 15451 genes, 77.1% | 2.11e-05 |  | [protein binding](http://amigo.geneontology.org/amigo/term/GO:0005515) | 631 out of 1674 genes, 37.7% | 4489 out of 15451 genes, 29.1% | 2.00e-13 |  | [protein binding](http://amigo.geneontology.org/amigo/term/GO:0005515) | 658 out of 1731 genes, 38.0% | 4489 out of 15451 genes, 29.1% | 4.43e-15 |
|  | [nucleic acid binding](http://amigo.geneontology.org/amigo/term/GO:0003676) | 113 out of 436 genes, 25.9% | 2561 out of 15451 genes, 16.6% | 7.03e-05 |  | [binding](http://amigo.geneontology.org/amigo/term/GO:0005488) | 1397 out of 1674 genes, 83.5% | 11911 out of 15451 genes, 77.1% | 3.57e-09 |  | [receptor binding](http://amigo.geneontology.org/amigo/term/GO:0005102) | 181 out of 1731 genes, 10.5% | 979 out of 15451 genes, 6.3% | 1.06e-09 |
|  | [transcription regulator activity](http://amigo.geneontology.org/amigo/term/GO:0030528) | 43 out of 436 genes, 9.9% | 757 out of 15451 genes, 4.9% | 0.00205 |  | [channel activity](http://amigo.geneontology.org/amigo/term/GO:0015267) | 87 out of 1674 genes, 5.2% | 395 out of 15451 genes, 2.6% | 2.66e-08 |  | [binding](http://amigo.geneontology.org/amigo/term/GO:0005488) | 1435 out of 1731 genes, 82.9% | 11911 out of 15451 genes, 77.1% | 9.19e-08 |
|  | [iron ion binding](http://amigo.geneontology.org/amigo/term/GO:0005506) | 18 out of 436 genes, 4.1% | 211 out of 15451 genes, 1.4% | 0.00634 |  | [passive transmembrane transporter activity](http://amigo.geneontology.org/amigo/term/GO:0022803) | 87 out of 1674 genes, 5.2% | 397 out of 15451 genes, 2.6% | 3.51e-08 |  | [identical protein binding](http://amigo.geneontology.org/amigo/term/GO:0042802) | 107 out of 1731 genes, 6.2% | 534 out of 15451 genes, 3.5% | 4.68e-07 |
|  | [myosin binding](http://amigo.geneontology.org/amigo/term/GO:0017022) | 4 out of 436 genes, 0.9% | 14 out of 15451 genes, 0.1% | 0.10197 |  | [ion channel activity](http://amigo.geneontology.org/amigo/term/GO:0005216) | 82 out of 1674 genes, 4.9% | 373 out of 15451 genes, 2.4% | 1.05e-07 |  | [sequence-specific DNA binding transcription factor activity](http://amigo.geneontology.org/amigo/term/GO:0003700) | 31 out of 1731 genes, 1.8% | 104 out of 15451 genes, 0.7% | 9.05e-05 |
|  | [sequence-specific DNA binding transcription factor activity](http://amigo.geneontology.org/amigo/term/GO:0003700) | 10 out of 436 genes, 2.3% | 104 out of 15451 genes, 0.7% | 0.14444 |  | [substrate-specific channel activity](http://amigo.geneontology.org/amigo/term/GO:0022838) | 82 out of 1674 genes, 4.9% | 373 out of 15451 genes, 2.4% | 1.05e-07 |  | [sequence-specific DNA binding RNA polymerase II transcription factor activity](http://amigo.geneontology.org/amigo/term/GO:0000981) | 28 out of 1731 genes, 1.6% | 94 out of 15451 genes, 0.6% | 0.00035 |
|  | [sequence-specific DNA binding](http://amigo.geneontology.org/amigo/term/GO:0043565) | 17 out of 436 genes, 3.9% | 257 out of 15451 genes, 1.7% | 0.21124 |  | [carbohydrate binding](http://amigo.geneontology.org/amigo/term/GO:0030246) | 84 out of 1674 genes, 5.0% | 392 out of 15451 genes, 2.5% | 2.41e-07 |  | [transmembrane transporter activity](http://amigo.geneontology.org/amigo/term/GO:0022857) | 149 out of 1731 genes, 8.6% | 918 out of 15451 genes, 5.9% | 0.00062 |
|  | [myosin heavy chain binding](http://amigo.geneontology.org/amigo/term/GO:0032036) | 2 out of 436 genes, 0.5% | 3 out of 15451 genes, 0.0% | 0.47712 |  | [gated channel activity](http://amigo.geneontology.org/amigo/term/GO:0022836) | 44 out of 1674 genes, 2.6% | 153 out of 15451 genes, 1.0% | 3.34e-07 |  | [substrate-specific transmembrane transporter activity](http://amigo.geneontology.org/amigo/term/GO:0022891) | 136 out of 1731 genes, 7.9% | 828 out of 15451 genes, 5.4% | 0.00097 |
|  | [structural molecule activity](http://amigo.geneontology.org/amigo/term/GO:0005198) | 21 out of 436 genes, 4.8% | 389 out of 15451 genes, 2.5% | 0.72490 |  | [pattern binding](http://amigo.geneontology.org/amigo/term/GO:0001871) | 50 out of 1674 genes, 3.0% | 188 out of 15451 genes, 1.2% | 4.79e-07 |  | [pattern binding](http://amigo.geneontology.org/amigo/term/GO:0001871) | 42 out of 1731 genes, 2.4% | 188 out of 15451 genes, 1.2% | 0.00379 |
|  | [protein dimerization activity](http://amigo.geneontology.org/amigo/term/GO:0046983) | 19 out of 436 genes, 4.4% | 340 out of 15451 genes, 2.2% | 0.76451 |  | [transporter activity](http://amigo.geneontology.org/amigo/term/GO:0005215) | 179 out of 1674 genes, 10.7% | 1082 out of 15451 genes, 7.0% | 1.03e-06 |  | [growth factor binding](http://amigo.geneontology.org/amigo/term/GO:0019838) | 25 out of 1731 genes, 1.4% | 89 out of 15451 genes, 0.6% | 0.00431 |
| Biological progress | [pattern specification process](http://amigo.geneontology.org/amigo/term/GO:0007389) | 67 out of 428 genes, 15.7% | 280 out of 15332 genes, 1.8% | 1.28e-40 |  | [anatomical structure development](http://amigo.geneontology.org/amigo/term/GO:0048856) | 592 out of 1671 genes, 35.4% | 3174 out of 15332 genes, 20.7% | 1.12e-46 |  | [anatomical structure development](http://amigo.geneontology.org/amigo/term/GO:0048856) | 606 out of 1734 genes, 34.9% | 3174 out of 15332 genes, 20.7% | 1.10e-45 |
|  | [regionalization](http://amigo.geneontology.org/amigo/term/GO:0003002) | 61 out of 428 genes, 14.3% | 238 out of 15332 genes, 1.6% | 1.34e-38 |  | [developmental process](http://amigo.geneontology.org/amigo/term/GO:0032502) | 666 out of 1671 genes, 39.9% | 3787 out of 15332 genes, 24.7% | 1.09e-44 |  | [multicellular organismal process](http://amigo.geneontology.org/amigo/term/GO:0032501) | 824 out of 1734 genes, 47.5% | 4866 out of 15332 genes, 31.7% | 6.20e-45 |
|  | [anatomical structure development](http://amigo.geneontology.org/amigo/term/GO:0048856) | 203 out of 428 genes, 47.4% | 3174 out of 15332 genes, 20.7% | 5.30e-33 |  | [multicellular organismal development](http://amigo.geneontology.org/amigo/term/GO:0007275) | 554 out of 1671 genes, 33.2% | 2954 out of 15332 genes, 19.3% | 1.86e-43 |  | [developmental process](http://amigo.geneontology.org/amigo/term/GO:0032502) | 686 out of 1734 genes, 39.6% | 3787 out of 15332 genes, 24.7% | 6.89e-45 |
|  | [developmental process](http://amigo.geneontology.org/amigo/term/GO:0032502) | 224 out of 428 genes, 52.3% | 3787 out of 15332 genes, 24.7% | 1.53e-32 |  | [system development](http://amigo.geneontology.org/amigo/term/GO:0048731) | 508 out of 1671 genes, 30.4% | 2629 out of 15332 genes, 17.1% | 1.01e-42 |  | [system development](http://amigo.geneontology.org/amigo/term/GO:0048731) | 519 out of 1734 genes, 29.9% | 2629 out of 15332 genes, 17.1% | 1.36e-41 |
|  | [anatomical structure morphogenesis](http://amigo.geneontology.org/amigo/term/GO:0009653) | 122 out of 428 genes, 28.5% | 1366 out of 15332 genes, 8.9% | 1.33e-29 |  | [multicellular organismal process](http://amigo.geneontology.org/amigo/term/GO:0032501) | 793 out of 1671 genes, 47.5% | 4866 out of 15332 genes, 31.7% | 1.11e-42 |  | [multicellular organismal development](http://amigo.geneontology.org/amigo/term/GO:0007275) | 563 out of 1734 genes, 32.5% | 2954 out of 15332 genes, 19.3% | 4.26e-41 |
|  | [multicellular organismal development](http://amigo.geneontology.org/amigo/term/GO:0007275) | 186 out of 428 genes, 43.5% | 2954 out of 15332 genes, 19.3% | 3.51e-28 |  | [anatomical structure morphogenesis](http://amigo.geneontology.org/amigo/term/GO:0009653) | 316 out of 1671 genes, 18.9% | 1366 out of 15332 genes, 8.9% | 2.56e-39 |  | [organ development](http://amigo.geneontology.org/amigo/term/GO:0048513) | 365 out of 1734 genes, 21.0% | 1794 out of 15332 genes, 11.7% | 1.82e-29 |
|  | [embryo development](http://amigo.geneontology.org/amigo/term/GO:0009790) | 81 out of 428 genes, 18.9% | 657 out of 15332 genes, 4.3% | 1.60e-27 |  | [organ development](http://amigo.geneontology.org/amigo/term/GO:0048513) | 357 out of 1671 genes, 21.4% | 1794 out of 15332 genes, 11.7% | 4.53e-30 |  | [tissue development](http://amigo.geneontology.org/amigo/term/GO:0009888) | 192 out of 1734 genes, 11.1% | 807 out of 15332 genes, 5.3% | 1.08e-21 |
|  | [system development](http://amigo.geneontology.org/amigo/term/GO:0048731) | 171 out of 428 genes, 40.0% | 2629 out of 15332 genes, 17.1% | 9.25e-27 |  | [pattern specification process](http://amigo.geneontology.org/amigo/term/GO:0007389) | 103 out of 1671 genes, 6.2% | 280 out of 15332 genes, 1.8% | 2.03e-27 |  | [cellular developmental process](http://amigo.geneontology.org/amigo/term/GO:0048869) | 354 out of 1734 genes, 20.4% | 1884 out of 15332 genes, 12.3% | 1.78e-21 |
|  | [embryonic morphogenesis](http://amigo.geneontology.org/amigo/term/GO:0048598) | 62 out of 428 genes, 14.5% | 393 out of 15332 genes, 2.6% | 2.75e-26 |  | [organ morphogenesis](http://amigo.geneontology.org/amigo/term/GO:0009887) | 156 out of 1671 genes, 9.3% | 554 out of 15332 genes, 3.6% | 3.71e-27 |  | [cell differentiation](http://amigo.geneontology.org/amigo/term/GO:0030154) | 294 out of 1734 genes, 17.0% | 1477 out of 15332 genes, 9.6% | 4.60e-21 |
|  | [multicellular organismal process](http://amigo.geneontology.org/amigo/term/GO:0032501) | 246 out of 428 genes, 57.5% | 4866 out of 15332 genes, 31.7% | 9.92e-26 |  | [regionalization](http://amigo.geneontology.org/amigo/term/GO:0003002) | 93 out of 1671 genes, 5.6% | 238 out of 15332 genes, 1.6% | 6.93e-27 |  | [anatomical structure morphogenesis](http://amigo.geneontology.org/amigo/term/GO:0009653) | 277 out of 1734 genes, 16.0% | 1366 out of 15332 genes, 8.9% | 7.12e-21 |
|  | [organ development](http://amigo.geneontology.org/amigo/term/GO:0048513) | 132 out of 428 genes, 30.8% | 1794 out of 15332 genes, 11.7% | 5.56e-24 |  | [cell differentiation](http://amigo.geneontology.org/amigo/term/GO:0030154) | 291 out of 1671 genes, 17.4% | 1477 out of 15332 genes, 9.6% | 9.91e-23 |  | [organ morphogenesis](http://amigo.geneontology.org/amigo/term/GO:0009887) | 146 out of 1734 genes, 8.4% | 554 out of 15332 genes, 3.6% | 2.34e-20 |
|  | [organ morphogenesis](http://amigo.geneontology.org/amigo/term/GO:0009887) | 69 out of 428 genes, 16.1% | 554 out of 15332 genes, 3.6% | 3.35e-23 |  | [cellular developmental process](http://amigo.geneontology.org/amigo/term/GO:0048869) | 341 out of 1671 genes, 20.4% | 1884 out of 15332 genes, 12.3% | 1.77e-20 |  | [biological regulation](http://amigo.geneontology.org/amigo/term/GO:0065007) | 887 out of 1734 genes, 51.2% | 6153 out of 15332 genes, 40.1% | 9.24e-20 |
|  | [tissue development](http://amigo.geneontology.org/amigo/term/GO:0009888) | 79 out of 428 genes, 18.5% | 807 out of 15332 genes, 5.3% | 4.34e-20 |  | [tissue development](http://amigo.geneontology.org/amigo/term/GO:0009888) | 184 out of 1671 genes, 11.0% | 807 out of 15332 genes, 5.3% | 2.80e-20 |  | [nervous system development](http://amigo.geneontology.org/amigo/term/GO:0007399) | 230 out of 1734 genes, 13.3% | 1082 out of 15332 genes, 7.1% | 1.84e-19 |
|  | [embryonic organ development](http://amigo.geneontology.org/amigo/term/GO:0048568) | 38 out of 428 genes, 8.9% | 200 out of 15332 genes, 1.3% | 4.15e-18 |  | [embryonic organ development](http://amigo.geneontology.org/amigo/term/GO:0048568) | 75 out of 1671 genes, 4.5% | 200 out of 15332 genes, 1.3% | 5.93e-20 |  | [regulation of biological process](http://amigo.geneontology.org/amigo/term/GO:0050789) | 794 out of 1734 genes, 45.8% | 5400 out of 15332 genes, 35.2% | 7.92e-19 |
|  | [embryonic organ morphogenesis](http://amigo.geneontology.org/amigo/term/GO:0048562) | 34 out of 428 genes, 7.9% | 173 out of 15332 genes, 1.1% | 1.77e-16 |  | [embryo development](http://amigo.geneontology.org/amigo/term/GO:0009790)) | 159 out of 1671 genes, 9.5% | 657 out of 15332 genes, 4.3% | 6.28e-20 |  | [regulation of cellular process](http://amigo.geneontology.org/amigo/term/GO:0050794) | 692 out of 1734 genes, 39.9% | 4563 out of 15332 genes, 29.8% | 1.25e-18 |
|  | [cell differentiation](http://amigo.geneontology.org/amigo/term/GO:0030154) | 101 out of 428 genes, 23.6% | 1477 out of 15332 genes, 9.6% | 7.18e-15 |  | [embryonic morphogenesis](http://amigo.geneontology.org/amigo/term/GO:0048598) | 110 out of 1671 genes, 6.6% | 393 out of 15332 genes, 2.6% | 3.77e-18 |  | [central nervous system development](http://amigo.geneontology.org/amigo/term/GO:0007417) | 133 out of 1734 genes, 7.7% | 537 out of 15332 genes, 3.5% | 9.20e-16 |
|  | [regulation of transcription from RNA polymerase II promoter](http://amigo.geneontology.org/amigo/term/GO:0006357) | 72 out of 428 genes, 16.8% | 869 out of 15332 genes, 5.7% | 5.68e-14 |  | [embryonic organ morphogenesis](http://amigo.geneontology.org/amigo/term/GO:0048562) | 66 out of 1671 genes, 3.9% | 173 out of 15332 genes, 1.1% | 8.75e-18 |  | [regulation of transcription from RNA polymerase II promoter](http://amigo.geneontology.org/amigo/term/GO:0006357) | 186 out of 1734 genes, 10.7% | 869 out of 15332 genes, 5.7% | 1.81e-15 |
|  | [cellular developmental process](http://amigo.geneontology.org/amigo/term/GO:0048869) | 115 out of 428 genes, 26.9% | 1884 out of 15332 genes, 12.3% | 1.08e-13 |  | [regulation of transcription from RNA polymerase II promoter](http://amigo.geneontology.org/amigo/term/GO:0006357) | 183 out of 1671 genes, 11.0% | 869 out of 15332 genes, 5.7% | 4.05e-16 |  | [cell communication](http://amigo.geneontology.org/amigo/term/GO:0007154) | 178 out of 1734 genes, 10.3% | 835 out of 15332 genes, 5.4% | 1.70e-14 |
|  | [tissue morphogenesis](http://amigo.geneontology.org/amigo/term/GO:0048729) | 39 out of 428 genes, 9.1% | 285 out of 15332 genes, 1.9% | 1.82e-13 |  | [cell communication](http://amigo.geneontology.org/amigo/term/GO:0007154) | 177 out of 1671 genes, 10.6% | 835 out of 15332 genes, 5.4% | 8.33e-16 |  | [response to stimulus](http://amigo.geneontology.org/amigo/term/GO:0050896) | 589 out of 1734 genes, 34.0% | 3898 out of 15332 genes, 25.4% | 4.14e-14 |
|  | [regulation of transcription, DNA- dependent](http://amigo.geneontology.org/amigo/term/GO:0006355) | 89 out of 428 genes, 20.8% | 1381 out of 15332 genes, 9.0% | 3.88e-11 |  | [regulation of multicellular organismal process](http://amigo.geneontology.org/amigo/term/GO:0051239) | 216 out of 1671 genes, 12.9% | 1103 out of 15332 genes, 7.2% | 1.47e-15 |  | [regulation of multicellular organismal process](http://amigo.geneontology.org/amigo/term/GO:0051239) | 216 out of 1734 genes, 12.5% | 1103 out of 15332 genes, 7.2% | 1.14e-13 |
|  | [regulation of transcription](http://amigo.geneontology.org/amigo/term/GO:0045449) | 89 out of 428 genes, 20.8% | 1385 out of 15332 genes, 9.0% | 4.60e-11 |  | [nervous system development](http://amigo.geneontology.org/amigo/term/GO:0007399) | 211 out of 1671 genes, 12.6% | 1082 out of 15332 genes, 7.1% | 6.59e-15 |  | [regulation of biosynthetic process](http://amigo.geneontology.org/amigo/term/GO:0009889) | 294 out of 1734 genes, 17.0% | 1654 out of 15332 genes, 10.8% | 1.74e-13 |
|  | [regulation of RNA metabolic process](http://amigo.geneontology.org/amigo/term/GO:0051252) | 89 out of 428 genes, 20.8% | 1402 out of 15332 genes, 9.1% | 9.36e-11 |  | [biological regulation](http://amigo.geneontology.org/amigo/term/GO:0065007) | 834 out of 1671 genes, 49.9% | 6153 out of 15332 genes, 40.1% | 1.42e-14 |  | [regulation of cellular biosynthetic process](http://amigo.geneontology.org/amigo/term/GO:0031326) | 287 out of 1734 genes, 16.6% | 1604 out of 15332 genes, 10.5% | 1.79e-13 |
|  | [nervous system development](http://amigo.geneontology.org/amigo/term/GO:0007399) | 75 out of 428 genes, 17.5% | 1082 out of 15332 genes, 7.1% | 1.64e-10 |  | [muscle system process](http://amigo.geneontology.org/amigo/term/GO:0003012) | 67 out of 1671 genes, 4.0% | 215 out of 15332 genes, 1.4% | 9.22e-13 |  | [response to external stimulus](http://amigo.geneontology.org/amigo/term/GO:0009605) | 188 out of 1734 genes, 10.8% | 925 out of 15332 genes, 6.0% | 3.35e-13 |
|  | [regulation of cellular biosynthetic process](http://amigo.geneontology.org/amigo/term/GO:0031326) | 96 out of 428 genes, 22.4% | 1604 out of 15332 genes, 10.5% | 2.78e-10 |  | [regulation of biological process](http://amigo.geneontology.org/amigo/term/GO:0050789) | 738 out of 1671 genes, 44.2% | 5400 out of 15332 genes, 35.2% | 1.64e-12 |  | [embryonic organ development](http://amigo.geneontology.org/amigo/term/GO:0048568) | 66 out of 1734 genes, 3.8% | 200 out of 15332 genes, 1.3% | 3.90e-13 |
|  | [regulation of nucleobase, nucleoside, nucleotide and nucleic acid metabolic process](http://amigo.geneontology.org/amigo/term/GO:0019219) | 103 out of 428 genes, 24.1% | 1795 out of 15332 genes, 11.7% | 4.15e-10 |  | [anatomical structure formation involved in morphogenesis](http://amigo.geneontology.org/amigo/term/GO:0048646) | 110 out of 1671 genes, 6.6% | 470 out of 15332 genes, 3.1% | 6.20e-12 |  | [regulation of developmental process](http://amigo.geneontology.org/amigo/term/GO:0050793) | 170 out of 1734 genes, 9.8% | 816 out of 15332 genes, 5.3% | 1.00e-12 |
