# Supplementary material for: Reduced H3K27me3 leads to abnormal Hox gene expression in neural tube defects
Source: Epigenetics Chromatin. 2019 Dec 19;12:76. doi: 10.1186/s13072-019-0318-1 (PMC6921514; doi:10.1186/s13072-019-0318-1)
Supplement: Supplementary file 3 — Additional file 3: Table S2. Enriched KEGG pathways of DEGs in Con-E8.5-vs-RA-E8.5, Con-E9.5-vs-RA-E9.5 and Con-E10.5-vs-RA-E10.5 comparisons. [file 13072_2019_318_MOESM3_ESM.docx]

**Table S2. List of pathways for differentially expressed genes (DEGs)**

| Pathway term | Pathway ID | # of DEGs | *P* value | FDR |
| --- | --- | --- | --- | --- |
| Con-E9.5-vs-RA-E9.5 |  |  |  |  |
| [ECM-receptor interaction](file:///F:\于娟\合同\华大\脑泡转录组数据\report\Transcriptome_resequencing_report\Files\BGI_result\AdvancedAnalysis\GeneDiffExp\Pathway\Brc9_5-VS-Bra9_5.htm#gene1) | ko04512 | 48 (2.68%) | 4.29E-08 | 5.29E-06 |
| [Axon guidance](file:///F:\于娟\合同\华大\脑泡转录组数据\report\Transcriptome_resequencing_report\Files\BGI_result\AdvancedAnalysis\GeneDiffExp\Pathway\Brc8_5-VS-Brc9_5.htm#gene2) | ko04360 | 63 (3.52%) | 4.38E-08 | 5.29E-06 |
| [Pathways in cancer](file:///F:\于娟\合同\华大\脑泡转录组数据\report\Transcriptome_resequencing_report\Files\BGI_result\AdvancedAnalysis\GeneDiffExp\Pathway\Brc9_5-VS-Bra9_5.htm#gene3) | ko05200 | 97 (5.42%) | 9.08E-08 | 7.32E-06 |
| [Neuroactive ligand-receptor interaction](file:///F:\于娟\合同\华大\脑泡转录组数据\report\Transcriptome_resequencing_report\Files\BGI_result\AdvancedAnalysis\GeneDiffExp\Pathway\Brc9_5-VS-Bra9_5.htm#gene4) | ko04080 | 64 (3.57%) | 2.25E-05 | 1.36E-03 |
| [Focal adhesion](file:///F:\于娟\合同\华大\脑泡转录组数据\report\Transcriptome_resequencing_report\Files\BGI_result\AdvancedAnalysis\GeneDiffExp\Pathway\Brc9_5-VS-Bra9_5.htm#gene5) | ko04510 | 70 (3.91%) | 4.10E-05 | 1.98E-03 |
| [Basal cell carcinoma](file:///F:\于娟\合同\华大\脑泡转录组数据\report\Transcriptome_resequencing_report\Files\BGI_result\AdvancedAnalysis\GeneDiffExp\Pathway\Brc9_5-VS-Bra9_5.htm#gene6) | ko05217 | 19 (1.06%) | 7.19E-05 | 2.90E-03 |
| [Hedgehog signaling pathway](file:///F:\于娟\合同\华大\脑泡转录组数据\report\Transcriptome_resequencing_report\Files\BGI_result\AdvancedAnalysis\GeneDiffExp\Pathway\Brc9_5-VS-Bra9_5.htm#gene8) | ko04340 | 18 (1.01%) | 2.84E-04 | 8.12E-03 |
| [Protein digestion and absorption](file:///F:\于娟\合同\华大\脑泡转录组数据\report\Transcriptome_resequencing_report\Files\BGI_result\AdvancedAnalysis\GeneDiffExp\Pathway\Brc9_5-VS-Bra9_5.htm#gene9) | ko04974 | 31 (1.73%) | 3.02E-04 | 8.12Ee-03 |
| [Cholinergic synapse](file:///F:\于娟\合同\华大\脑泡转录组数据\report\Transcriptome_resequencing_report\Files\BGI_result\AdvancedAnalysis\GeneDiffExp\Pathway\Brc9_5-VS-Bra9_5.htm#gene10) | ko04725 | 29 (1.62%) | 3.58E-04 | 8.67E-03 |
| [Melanogenesis](file:///F:\于娟\合同\华大\脑泡转录组数据\report\Transcriptome_resequencing_report\Files\BGI_result\AdvancedAnalysis\GeneDiffExp\Pathway\Brc8_5-VS-Brc9_5.htm#gene10) | ko04916 | 27 (1.51%) | 5.43E-04 | 1.20E-02 |
| [Transcriptional misregulation in cancer](file:///F:\于娟\合同\华大\脑泡转录组数据\report\Transcriptome_resequencing_report\Files\BGI_result\AdvancedAnalysis\GeneDiffExp\Pathway\Brc9_5-VS-Bra9_5.htm#gene12) | ko05202 | 60 (3.35%) | 6.54E-04 | 1.32E-02 |
| [Dilated cardiomyopathy](file:///F:\于娟\合同\华大\脑泡转录组数据\report\Transcriptome_resequencing_report\Files\BGI_result\AdvancedAnalysis\GeneDiffExp\Pathway\Brc9_5-VS-Bra9_5.htm#gene13) | ko05414 | 48 (2.68%) | 7.25E-04 | 1.35E-02 |
| [Hypertrophic cardiomyopathy (HCM)](file:///F:\于娟\合同\华大\脑泡转录组数据\report\Transcriptome_resequencing_report\Files\BGI_result\AdvancedAnalysis\GeneDiffExp\Pathway\Brc9_5-VS-Bra9_5.htm#gene14) | ko05410 | 47 (2.62%) | 1.03E-03 | 1.78Ee-02 |
| [Arginine and proline metabolism](file:///F:\于娟\合同\华大\脑泡转录组数据\report\Transcriptome_resequencing_report\Files\BGI_result\AdvancedAnalysis\GeneDiffExp\Pathway\Brc9_5-VS-Bra9_5.htm#gene15) | ko00330 | 20 (1.12%) | 1.19E-03 | 1.90E-02 |
| [Calcium signaling pathway](file:///F:\于娟\合同\华大\脑泡转录组数据\report\Transcriptome_resequencing_report\Files\BGI_result\AdvancedAnalysis\GeneDiffExp\Pathway\Brc9_5-VS-Bra9_5.htm#gene16) | ko04020 | 41 (2.29%) | 1.25E-03 | 1.90E-02 |
| [Nicotine addiction](file:///F:\于娟\合同\华大\脑泡转录组数据\report\Transcriptome_resequencing_report\Files\BGI_result\AdvancedAnalysis\GeneDiffExp\Pathway\Brc9_5-VS-Bra9_5.htm#gene17) | ko05033 | 13 (0.73%) | 1.41E-03 | 2.01E-02 |
| [Systemic lupus erythematosus](file:///F:\于娟\合同\华大\脑泡转录组数据\report\Transcriptome_resequencing_report\Files\BGI_result\AdvancedAnalysis\GeneDiffExp\Pathway\Brc9_5-VS-Bra9_5.htm#gene19) | ko05322 | 33 (1.84%) | 1.82E-03 | 2.32E-02 |
| [Cell adhesion molecules (CAMs)](file:///F:\于娟\合同\华大\脑泡转录组数据\report\Transcriptome_resequencing_report\Files\BGI_result\AdvancedAnalysis\GeneDiffExp\Pathway\Brc9_5-VS-Bra9_5.htm#gene20) | ko04514 | 38 (2.12%) | 2.26E-03 | 2.73E-02 |
| [Glycosphingolipid biosynthesis - globo series](file:///F:\于娟\合同\华大\脑泡转录组数据\report\Transcriptome_resequencing_report\Files\BGI_result\AdvancedAnalysis\GeneDiffExp\Pathway\Brc9_5-VS-Bra9_5.htm#gene21) | ko00603 | 7 (0.39%) | 2.40E-03 | 2.77E-02 |
| [Glycolysis / Gluconeogenesis](file:///F:\于娟\合同\华大\脑泡转录组数据\report\Transcriptome_resequencing_report\Files\BGI_result\AdvancedAnalysis\GeneDiffExp\Pathway\Brc9_5-VS-Bra9_5.htm#gene22) | ko00010 | 19 (1.06%) | 2.92E-03 | 3.21E-02 |
| [Retrograde endocannabinoid signaling](file:///F:\于娟\合同\华大\脑泡转录组数据\report\Transcriptome_resequencing_report\Files\BGI_result\AdvancedAnalysis\GeneDiffExp\Pathway\Brc9_5-VS-Bra9_5.htm#gene23) | ko04723 | 24 (1.34%) | 3.26E-03 | 3.37E-02 |
| [Aldosterone-regulated sodium reabsorption](file:///F:\于娟\合同\华大\脑泡转录组数据\report\Transcriptome_resequencing_report\Files\BGI_result\AdvancedAnalysis\GeneDiffExp\Pathway\Brc9_5-VS-Bra9_5.htm#gene24) | ko04960 | 15 (0.84%) | 3.34E-03 | 3.37E-02 |
| [Cytokine-cytokine receptor interaction](file:///F:\于娟\合同\华大\脑泡转录组数据\report\Transcriptome_resequencing_report\Files\BGI_result\AdvancedAnalysis\GeneDiffExp\Pathway\Brc9_5-VS-Bra9_5.htm#gene25) | ko04060 | 53 (2.96%) | 4.53E-03 | 4.39E-02 |
| [Proximal tubule bicarbonate reclamation](file:///F:\于娟\合同\华大\脑泡转录组数据\report\Transcriptome_resequencing_report\Files\BGI_result\AdvancedAnalysis\GeneDiffExp\Pathway\Brc9_5-VS-Bra9_5.htm#gene26) | ko04964 | 44(0.26%) | 5.28E-03 | 4.85E-02 |
| [Melanoma](file:///F:\于娟\合同\华大\脑泡转录组数据\report\Transcriptome_resequencing_report\Files\BGI_result\AdvancedAnalysis\GeneDiffExp\Pathway\Brc9_5-VS-Bra9_5.htm#gene27) | ko05218 | 96 (0.57%) | 5.41E-03 | 4.85E-02 |
| Con-E10.5-vs-RA-E10.5 |  |  |  |  |
| [ECM-receptor interaction](file:///F:\于娟\合同\华大\脑泡转录组数据\report\Transcriptome_resequencing_report\Files\BGI_result\AdvancedAnalysis\GeneDiffExp\Pathway\Brc10_5-VS-Bra10_5.htm#gene1) | ko04512 | 53 (2.85%) | 7.43E-10 | 1.80E-07 |
| [Cytokine-cytokine receptor interaction](file:///F:\于娟\合同\华大\脑泡转录组数据\report\Transcriptome_resequencing_report\Files\BGI_result\AdvancedAnalysis\GeneDiffExp\Pathway\Brc10_5-VS-Bra10_5.htm#gene2) | ko04060 | 74 (3.98%) | 1.77E-08 | 2.14E-06 |
| [Neuroactive ligand-receptor interaction](file:///F:\于娟\合同\华大\脑泡转录组数据\report\Transcriptome_resequencing_report\Files\BGI_result\AdvancedAnalysis\GeneDiffExp\Pathway\Brc10_5-VS-Bra10_5.htm#gene3) | ko04080 | 75 (4.03%) | 3.11E-08 | 2.38E-06 |
| [Pathways in cancer](file:///F:\于娟\合同\华大\脑泡转录组数据\report\Transcriptome_resequencing_report\Files\BGI_result\AdvancedAnalysis\GeneDiffExp\Pathway\Brc10_5-VS-Bra10_5.htm#gene4) | ko05200 | 101 (5.43%) | 3.93E-08 | 2.38E-06 |
| [Glycolysis / Gluconeogenesis](file:///F:\于娟\合同\华大\脑泡转录组数据\report\Transcriptome_resequencing_report\Files\BGI_result\AdvancedAnalysis\GeneDiffExp\Pathway\Brc10_5-VS-Bra10_5.htm#gene5) | ko00010 | 28 (1.51%) | 2.72E-07 | 1.32E-05 |
| [Cell adhesion molecules (CAMs)](file:///F:\于娟\合同\华大\脑泡转录组数据\report\Transcriptome_resequencing_report\Files\BGI_result\AdvancedAnalysis\GeneDiffExp\Pathway\Brc10_5-VS-Bra10_5.htm#gene6) | ko04514 | 50 (2.69%) | 6.91E-07 | 2.79E-05 |
| [Basal cell carcinoma](file:///F:\于娟\合同\华大\脑泡转录组数据\report\Transcriptome_resequencing_report\Files\BGI_result\AdvancedAnalysis\GeneDiffExp\Pathway\Brc10_5-VS-Bra10_5.htm#gene7) | ko05217 | 20 (1.08%) | 3.56E-05 | 1.20E-03 |
| [Focal adhesion](file:///F:\于娟\合同\华大\脑泡转录组数据\report\Transcriptome_resequencing_report\Files\BGI_result\AdvancedAnalysis\GeneDiffExp\Pathway\Brc10_5-VS-Bra10_5.htm#gene8) | ko04510 | 72 (3.87%) | 4.19E-05 | 1.20E-03 |
| [Hedgehog signaling pathway](file:///F:\于娟\合同\华大\脑泡转录组数据\report\Transcriptome_resequencing_report\Files\BGI_result\AdvancedAnalysis\GeneDiffExp\Pathway\Brc10_5-VS-Bra10_5.htm#gene9) | ko04340 | 20 (1.08%) | 4.47E-05 | 1.20E-03 |
| [Melanoma](file:///F:\于娟\合同\华大\脑泡转录组数据\report\Transcriptome_resequencing_report\Files\BGI_result\AdvancedAnalysis\GeneDiffExp\Pathway\Brc10_5-VS-Bra10_5.htm#gene10) | ko05218 | 22 (1.18%) | 6.32E-04 | 1.53E-02 |
| [Jak-STAT signaling pathway](file:///F:\于娟\合同\华大\脑泡转录组数据\report\Transcriptome_resequencing_report\Files\BGI_result\AdvancedAnalysis\GeneDiffExp\Pathway\Brc10_5-VS-Bra10_5.htm#gene11) | ko04630 | 43 (2.31%) | 8.31E-04 | 1.69E-02 |
| [Hematopoietic cell lineage](file:///F:\于娟\合同\华大\脑泡转录组数据\report\Transcriptome_resequencing_report\Files\BGI_result\AdvancedAnalysis\GeneDiffExp\Pathway\Brc10_5-VS-Bra10_5.htm#gene12) | ko04640 | 28 (1.51%) | 8.99E-04 | 1.69E-02 |
| [Melanogenesis](file:///F:\于娟\合同\华大\脑泡转录组数据\report\Transcriptome_resequencing_report\Files\BGI_result\AdvancedAnalysis\GeneDiffExp\Pathway\Brc10_5-VS-Bra10_5.htm#gene13) | ko04916 | 27 (1.45%) | 9.63E-04 | 1.69E-02 |
| [Staphylococcus aureus infection](file:///F:\于娟\合同\华大\脑泡转录组数据\report\Transcriptome_resequencing_report\Files\BGI_result\AdvancedAnalysis\GeneDiffExp\Pathway\Brc10_5-VS-Bra10_5.htm#gene14) | ko05150 | 24 (1.29%) | 1.02E-03 | 1.69E-02 |
| [MAPK signaling pathway](file:///F:\于娟\合同\华大\脑泡转录组数据\report\Transcriptome_resequencing_report\Files\BGI_result\AdvancedAnalysis\GeneDiffExp\Pathway\Brc10_5-VS-Bra10_5.htm#gene15) | ko04010 | 65 (3.5%) | 1.06E-03 | 1.69E-02 |
| [Alanine, aspartate and glutamate metabolism](file:///F:\于娟\合同\华大\脑泡转录组数据\report\Transcriptome_resequencing_report\Files\BGI_result\AdvancedAnalysis\GeneDiffExp\Pathway\Brc10_5-VS-Bra10_5.htm#gene16) | ko00250 | 12 (0.65%) | 1.16E-03 | 1.69E-02 |
| [Complement and coagulation cascades](file:///F:\于娟\合同\华大\脑泡转录组数据\report\Transcriptome_resequencing_report\Files\BGI_result\AdvancedAnalysis\GeneDiffExp\Pathway\Brc10_5-VS-Bra10_5.htm#gene17) | ko04610 | 34 (1.83%) | 1.19E-03 | 1.69E-02 |
| [Renal cell carcinoma](file:///F:\于娟\合同\华大\脑泡转录组数据\report\Transcriptome_resequencing_report\Files\BGI_result\AdvancedAnalysis\GeneDiffExp\Pathway\Brc10_5-VS-Bra10_5.htm#gene18) | ko05211 | 22 (1.18%) | 1.69E-03 | 2.28E-02 |
| [Axon guidance](file:///F:\于娟\合同\华大\脑泡转录组数据\report\Transcriptome_resequencing_report\Files\BGI_result\AdvancedAnalysis\GeneDiffExp\Pathway\Brc10_5-VS-Bra10_5.htm#gene19) |  | 49 (2.64%) | 2.25E-03 | 2.87E-02 |
| [Cholinergic synapse](file:///F:\于娟\合同\华大\脑泡转录组数据\report\Transcriptome_resequencing_report\Files\BGI_result\AdvancedAnalysis\GeneDiffExp\Pathway\Brc10_5-VS-Bra10_5.htm#gene20) | ko04725 | 27 (1.45%) | 2.95E-03 | 3.57E-02 |
| [Arrhythmogenic right ventricular cardiomyopathy (ARVC)](file:///F:\于娟\合同\华大\脑泡转录组数据\report\Transcriptome_resequencing_report\Files\BGI_result\AdvancedAnalysis\GeneDiffExp\Pathway\Brc10_5-VS-Bra10_5.htm#gene22) | ko05412 | 26 (1.4%) | 3.57E-03 | 3.93E-02 |
| Hypertrophic cardiomyopathy (HCM) | ko05410 | 46 (2.47%) | 3.73E-03 | 3.93E-02 |
| [Leukocyte transendothelial migration](file:///F:\于娟\合同\华大\脑泡转录组数据\report\Transcriptome_resequencing_report\Files\BGI_result\AdvancedAnalysis\GeneDiffExp\Pathway\Brc10_5-VS-Bra10_5.htm#gene24) | ko04670 | 41 (2.21%) | 3.98E-03 | 3.96E-02 |
| [Transcriptional misregulation in cancer](file:///F:\于娟\合同\华大\脑泡转录组数据\report\Transcriptome_resequencing_report\Files\BGI_result\AdvancedAnalysis\GeneDiffExp\Pathway\Brc10_5-VS-Bra10_5.htm#gene25) | ko05202 | 58 (3.12%) | 4.13E-03 | 3.96E-02 |
| [Rheumatoid arthritis](file:///F:\于娟\合同\华大\脑泡转录组数据\report\Transcriptome_resequencing_report\Files\BGI_result\AdvancedAnalysis\GeneDiffExp\Pathway\Brc10_5-VS-Bra10_5.htm#gene27) | ko05323 | 22 (1.18%) | 4.49E-03 | 3.96E-02 |
| [Dilated cardiomyopathy](file:///F:\于娟\合同\华大\脑泡转录组数据\report\Transcriptome_resequencing_report\Files\BGI_result\AdvancedAnalysis\GeneDiffExp\Pathway\Brc10_5-VS-Bra10_5.htm#gene28) | ko05414 | 46 (2.47%) | 4.58E-03 | 3.96E-02 |
| [Glycine, serine and threonine metabolism](file:///F:\于娟\合同\华大\脑泡转录组数据\report\Transcriptome_resequencing_report\Files\BGI_result\AdvancedAnalysis\GeneDiffExp\Pathway\Brc10_5-VS-Bra10_5.htm#gene29) | ko00260 | 14 (0.75%) | 4.89E-03 | 4.08E-02 |
| [Nicotine addiction](file:///F:\于娟\合同\华大\脑泡转录组数据\report\Transcriptome_resequencing_report\Files\BGI_result\AdvancedAnalysis\GeneDiffExp\Pathway\Brc10_5-VS-Bra10_5.htm#gene30) | ko05033 | 12 (0.65%) | 5.90E-03 | 4.76E-02 |
