# Supplementary material for: Reduced H3K27me3 leads to abnormal Hox gene expression in neural tube defects
Source: Epigenetics Chromatin. 2019 Dec 19;12:76. doi: 10.1186/s13072-019-0318-1 (PMC6921514; doi:10.1186/s13072-019-0318-1)

Figure S2

A

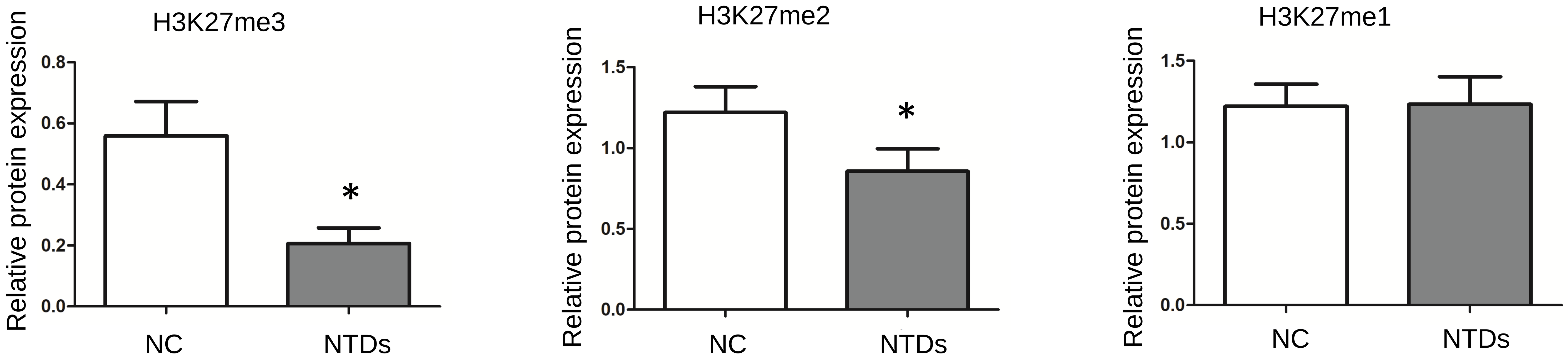

B

Peak statistics of mouse embryos of E10.5 used for ChIP-seq

Sample ID	Peak Number	Total Length	Average Length	Total Tag Depth	Average Tag Depth	Genome Rate (%)
Normal	52,759	9,336,717	176	587,746	11	0.34
NTDs	13,059	2,046,083	156	140,562	10	0.07

C

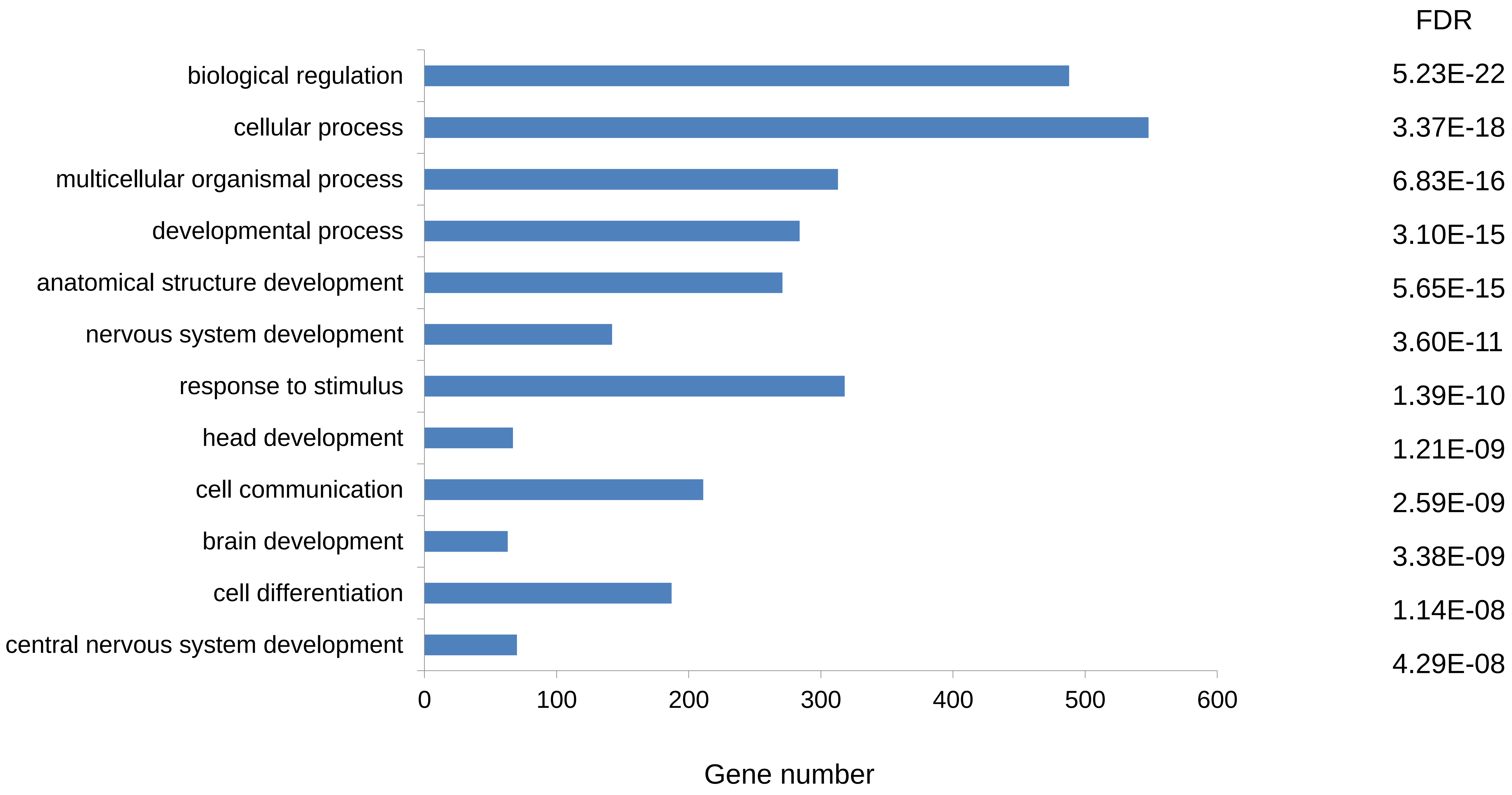

Supplement: Supplementary file 4 — Additional file 4: Figure S2. H3K27me3/2/1 analysis in mouse NTD embryos of E10.5. A. Relative protein expression of H3K27me3, H3K27me2 and H3K27me1 in mouse NTDs embryo. Data are shown as the mean (SD; n= 3). *P < 0.05. B. Peak statistics of mouse embryos used for ChIP-seq. C. GO analysis of differential peak related gene. [file 13072_2019_318_MOESM4_ESM.pdf]
