# Supplementary material for: Reduced H3K27me3 leads to abnormal Hox gene expression in neural tube defects
Source: Epigenetics Chromatin. 2019 Dec 19;12:76. doi: 10.1186/s13072-019-0318-1 (PMC6921514; doi:10.1186/s13072-019-0318-1)
Supplement: Supplementary file 8 — Additional file 8: Figure S5. HOX gene expression in human spinal bifida, hydrocephaly and encephalocele. A. HOX genes were detected with NanoString in the spinal cord from human spinal bifida and normal cases. Data are shown as the mean (SD; n= 10). P < 0.05 indicates statistical significance. B. HOX genes were detected with NanoString in the brain tissues from human hydrocephaly and normal cases. Data are shown as the mean (SD; n= 10). P < 0.05 indicates statistical significance. C. HOX genes were detected with NanoString in the brain tissues from human encephalocele and normal cases. Data are shown as the mean (SD; n= 9). P < 0.05 indicates statistical significance. D. Whole genome sequencing of 100 human NTDs samples. E, F. Variant distribution allele frequency of 10 HOX genes in 100 human NTDs samples. G. The number of variants by minor allele frequency (MAF) in 100 human NTDs samples. H, I. Variant rate of 10 HOX genes in 100 human NTDs samples. [file 13072_2019_318_MOESM8_ESM.pdf]

Figure S5

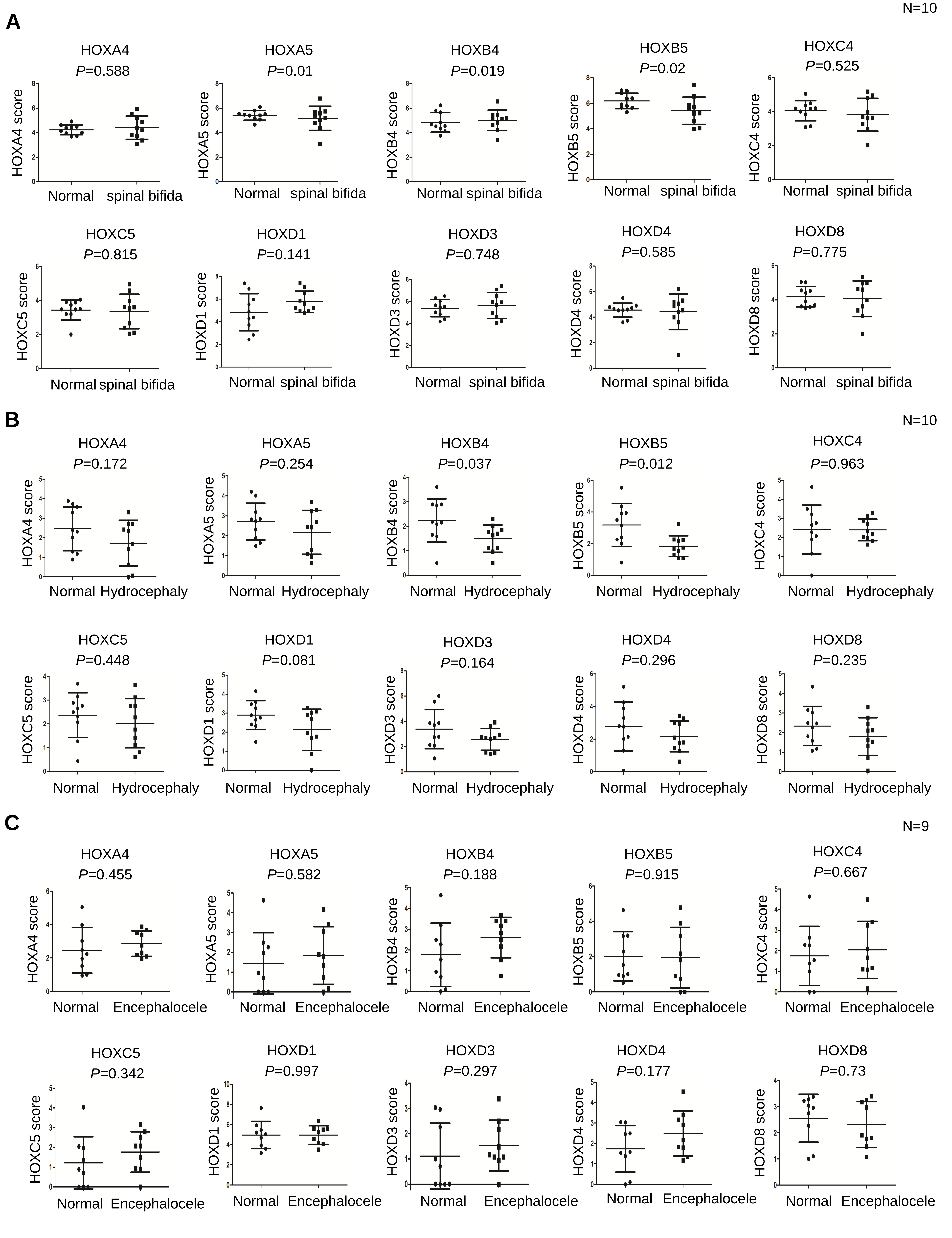

A
N=10
HOXA4
P=0.588
HOXA4 score
HOXA5
P=0.01
HOXA5 score
HOXB4
P=0.019
HOXB4 score
HOXB5
P=0.02
HOXB5 score
HOXC4
P=0.525
HOXC4 score
HOXC5
P=0.815
HOXC5 score
HOXD1
P=0.141
HOXD1 score
HOXD3
P=0.748
HOXD3 score
HOXD4
P=0.585
HOXD4 score
HOXD8
P=0.775
HOXD8 score
Normal
spinal bifida
B
N=10
HOXA4
P=0.172
HOXA4 score
HOXA5
P=0.254
HOXA5 score
HOXB4
P=0.037
HOXB4 score
HOXB5
P=0.012
HOXB5 score
HOXC4
P=0.963
HOXC4 score
HOXC5
P=0.448
HOXC5 score
HOXD1
P=0.081
HOXD1 score
HOXD3
P=0.164
HOXD3 score
HOXD4
P=0.296
HOXD4 score
HOXD8
P=0.235
HOXD8 score
Normal
Hydrocephaly
C
N=9
HOXA4
P=0.455
HOXA4 score
HOXA5
P=0.582
HOXA5 score
HOXB4
P=0.188
HOXB4 score
HOXB5
P=0.915
HOXB5 score
HOXC4
P=0.667
HOXC4 score
HOXC5
P=0.342
HOXC5 score
HOXD1
P=0.997
HOXD1 score
HOXD3
P=0.297
HOXD3 score
HOXD4
P=0.177
HOXD4 score
HOXD8
P=0.73
HOXD8 score
Normal
Encephalocele

D

100 NTD sample

↓

Whole genome sequencing

↓

HOX gene Mutation

Average Coverage	Mean depth
99.10%	39×

E

HOX genes	A4	A5	B4	B5	C4	C5	D1	D3	D4	D8	Count
Variant Type											
Intron	1	1	1	2	112	1	1	143	0	0	262
3'UTR	4	4	14	4	11	5	3	3	2	4	54
Upstream	0	0	0	0	0	0	0	0	0	0	0
Downstream	0	0	0	0	0	0	0	0	0	0	0
Non coding transcript exon	0	0	0	0	2	0	0	2	0	0	4
Missense	8	1	1	0	2	0	2	0	1	5	20
Synonymous	1	0	3	1	0	0	1	3	0	2	11
5'UTR	0	0	12	6	3	0	1	2	1	2	27
Splice region	1	0	0	0	1	0	0	0	0	0	2
Intrame del	0	0	0	0	0	0	0	0	1	3	4
Frame shift	0	0	0	0	0	0	0	0	0	0	0
Splicing donor	0	0	0	0	0	0	0	0	0	0	0
Splicing acceptor	0	0	0	0	0	0	0	0	0	0	0
Initiator codon	0	0	0	0	0	0	0	0	0	0	0
Stop gained	0	0	0	0	0	0	0	0	0	0	0
Total	15	6	31	13	131	6	8	153	5	16	384

F

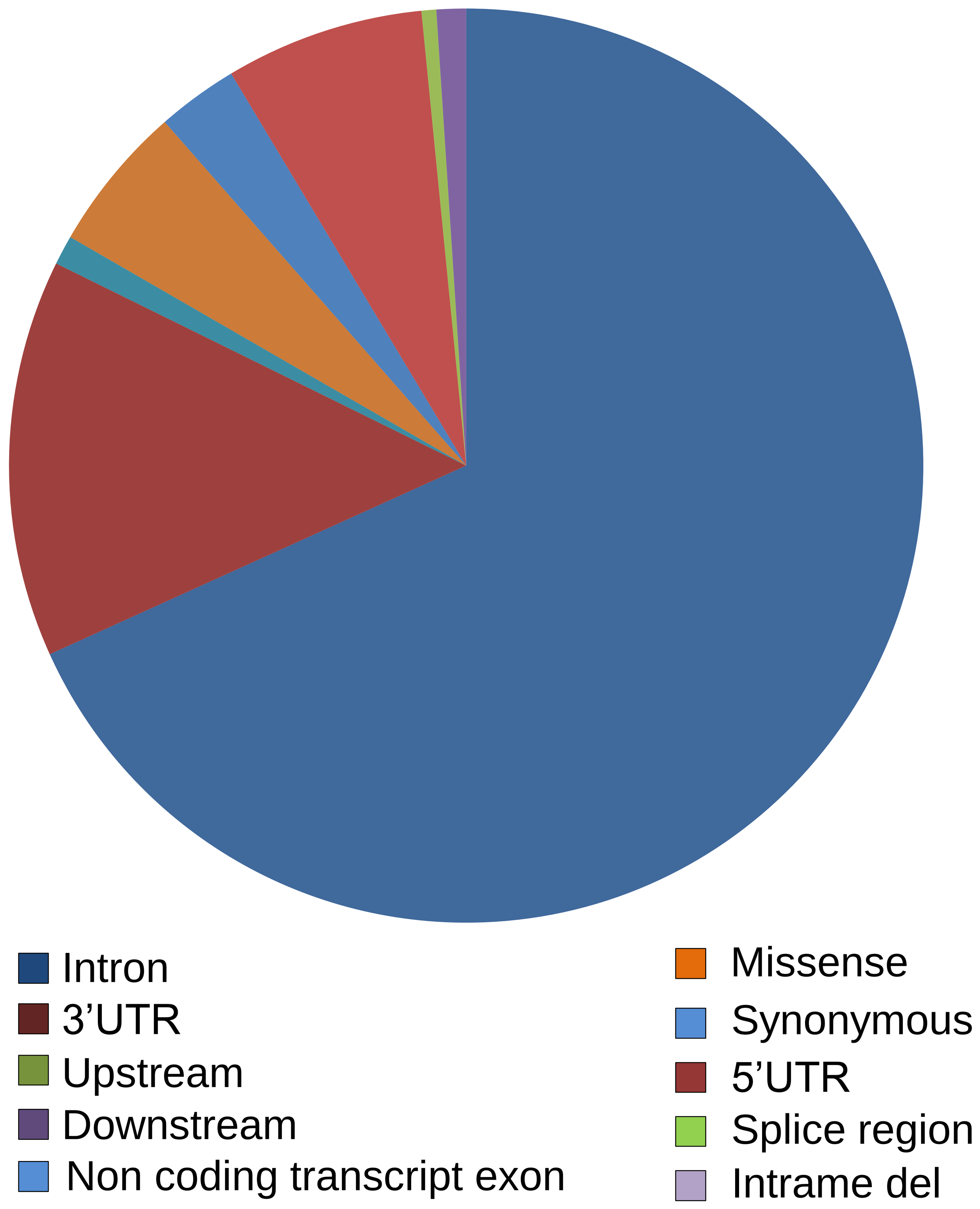

G

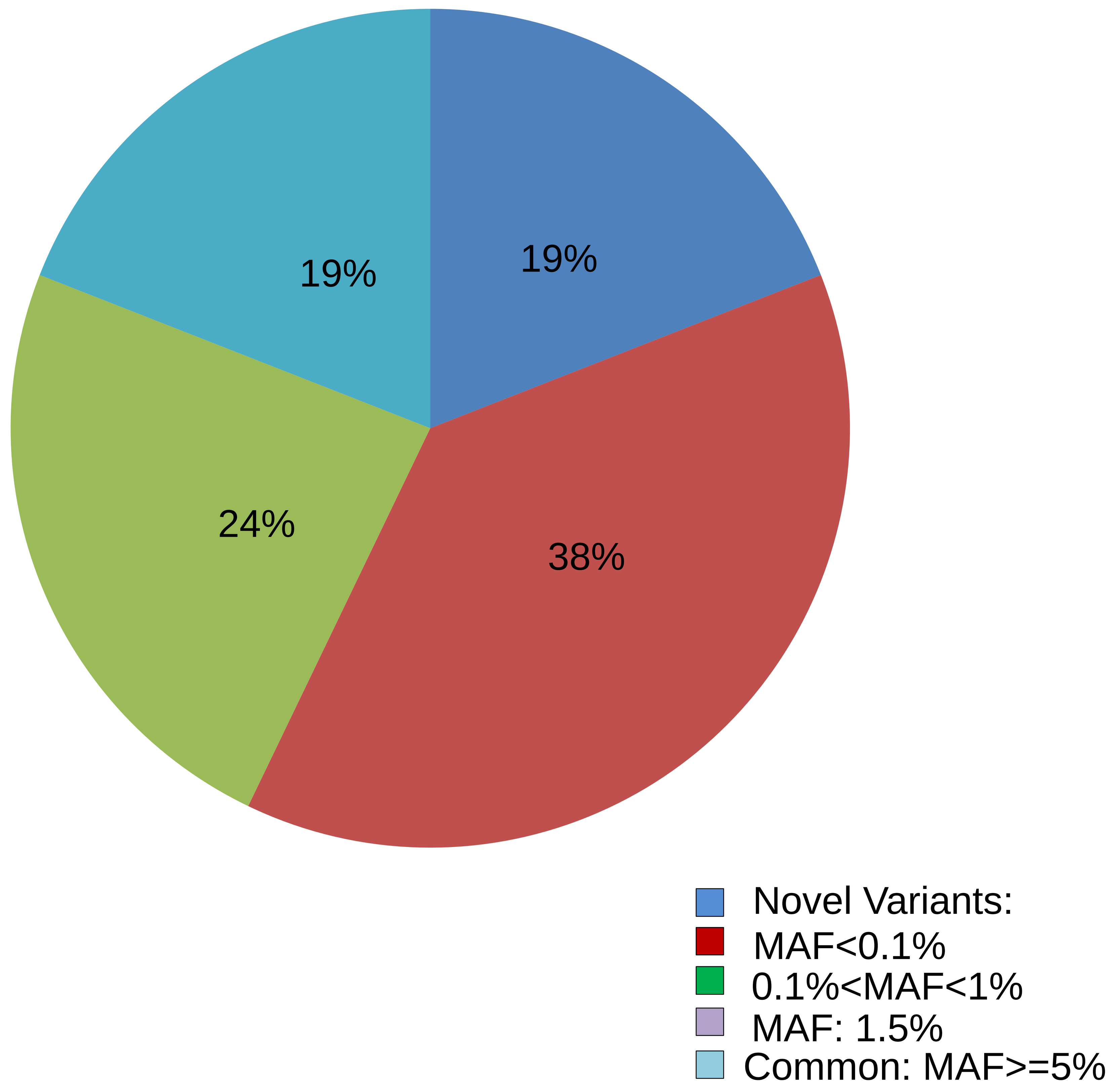

H

10 HOX genes

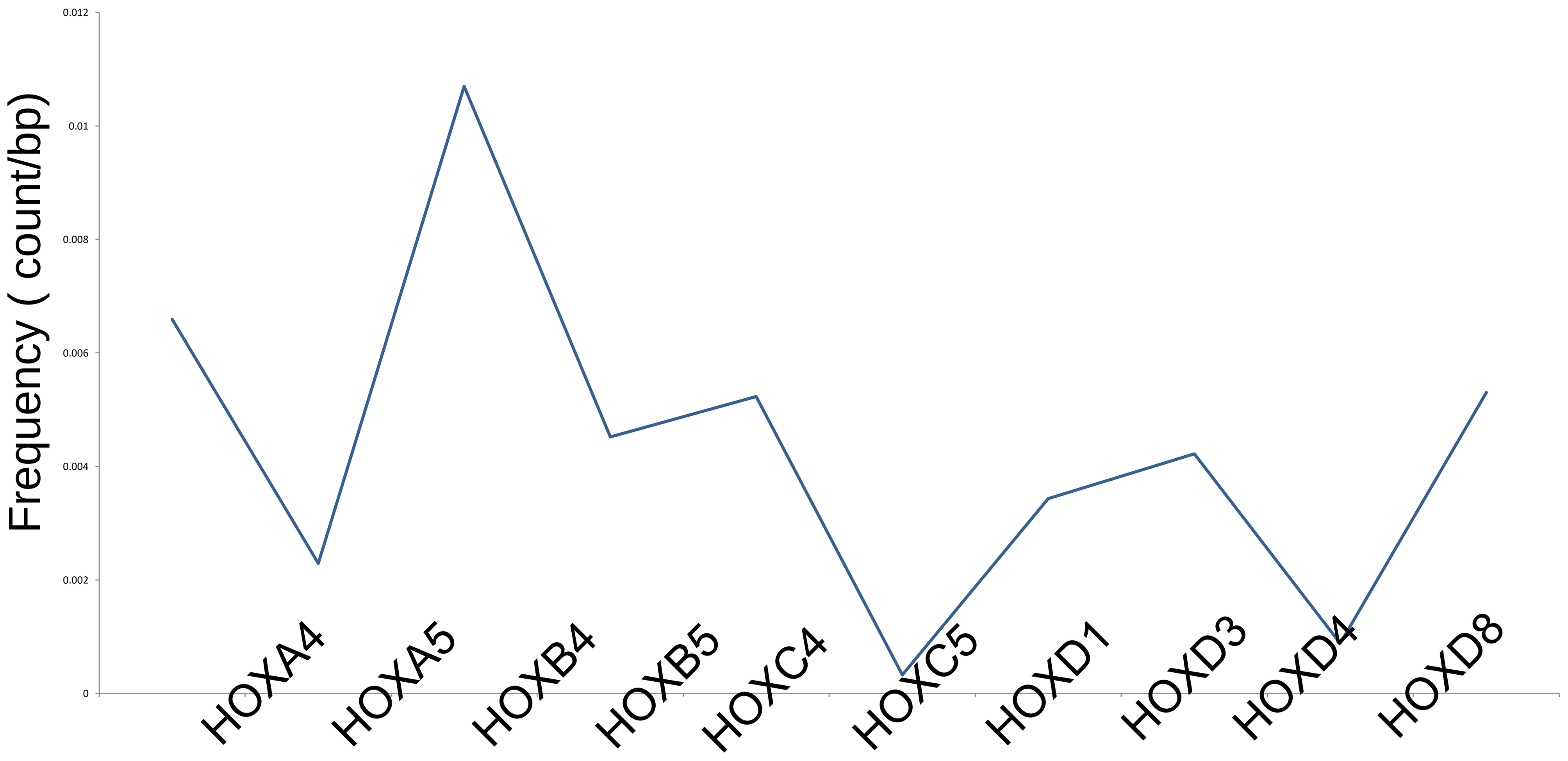

I

	Genes	Mut	Exon(missense)	Intron	5'-UTR	3-'UTR
HOX genes	10	384	35(20)	262	27	54
	Average/gene	38.4	3.5(2)	26.2	2.7	5.4

Sample	Genes	Variants number	Frequency/1000bp	Freq/1000bp MAF<0.1%/1000bp
100 NTD	10 HOX	384	2.2	2.4
