# Supplementary material for: Reduced H3K27me3 leads to abnormal Hox gene expression in neural tube defects
Source: Epigenetics Chromatin. 2019 Dec 19;12:76. doi: 10.1186/s13072-019-0318-1 (PMC6921514; doi:10.1186/s13072-019-0318-1)
Supplement: Supplementary file 9 — Additional file 9: Table S4. Information of human NTDs samples. The human cases with anencephaly, spinal bifida, hydrocephaly and encephalocele involved in this study. Gender, gestational age, sample type and NTDs phenotype are listed. [file 13072_2019_318_MOESM9_ESM.docx]

**Table S4 Information of human sample**

| No. of pairs | Sample No. | |  | Characteristic | | | NTDs Phenotype |
| --- | --- | --- | --- | --- | --- | --- | --- |
|  | Normal | Case |  | Gender | Gestational weeks | Sample type |  |
| 1 | A2067 | A1807 |  | Male | 21 | Brain | Anencephaly |
| 2 | A2061 | A2858 |  | Female | 20 | Brain | Anencephaly |
| 3 | A2412 | A2872 |  | Female | 20 | Brain | Craniorachischisis |
| 4 | A1895 | A2542 |  | Female | 25 | Brain | Anencephaly+ spina bifida |
| 5 | A2189 | A2497 |  | Female | 15 | Brain | Craniorachischisis |
| 6 | A1649 | A2920 |  | Female | 18 | Brain | Anencephaly |
| 7 | A2223 | A2633 |  | Female | 24 | Brain | Anencephaly |
| 8 | A1859 | A2776 |  | Male | 16 | Brain | Craniorachischisis |
| 9 | A1515 | A2734 |  | Female | 18 | Brain | Anencephaly+ spina bifida |
| 10 | A2188 | A2595 |  | Female | 16 | Brain | Anencephaly |
| 11 | A2279 | A2336 |  | Female | 16 | Spinal | Spina bifida |
| 12 | A1748 | A2155 |  | Female | 18 | Spinal | Craniorachischisis |
| 13 | A2856 | A2397 |  | Male | 19 | Spinal | Craniorachischisis |
| 14 | A2448 | A2671 |  | Male | 20 | Spinal | Anencephaly+ spina bifida |
| 15 | A1677 | A2875 |  | Female | 21 | Spinal | Spina bifida |
| 16 | A2731 | A2415 |  | Male | 23 | Spinal | Anencephaly+ spina bifida |
| 17 | A1525 | A2867 |  | Male | 24 | Spinal | Spina bifida |
| 18 | A2337 | A2889 |  | Female | 24 | Spinal | Anencephaly+ spina bifida |
| 19 | A1586 | A1490 |  | Male | 25 | Spinal | Spina bifida |
| 20 | A2132 | A2542 |  | Female | 25 | Spinal | Spina bifida |
| 21 | A1676 | A2875 |  | Female | 21 | Brain | Hydrocephaly+spina bifida |
| 22 | A1546 | A2193 |  | Female | 25 | Brain | Hydrocephaly+spina bifida |
| 23 | A1677 | A2709 |  | Female | 21 | Brain | Hydrocephaly+spina bifida |
| 24 | A2225 | A2867 |  | Male | 24 | Brain | Hydrocephaly+spina bifida |
| 25 | A1933 | A2691 |  | Male | 22 | Brain | Hydrocephaly+spina bifida |
| 26 | A2364 | A2162 |  | Male | 21 | Brain | Hydrocephaly+spina bifida |
| 27 | A2446 | A2738 |  | Female | 20 | Brain | Hydrocephaly+spina bifida |
| 28 | A2454 | A2888 |  | Female | 23 | Brain | Hydrocephaly+spina bifida |
| 29 | A2678 | A2889 |  | Female | 24 | Brain | Hydrocephaly+spina bifida |
| 30 | A1768 | A1490 |  | Male | 25 | Brain | Hydrocephaly+spina bifida |
| 31 | A1534 | A2611 |  | Male | 30 | Brain | Encephalocele |
| 32 | A1374 | A2666 |  | Female | 20 | Brain | Encephalocele |
| 33 | A2238 | A2643 |  | Female | 20 | Brain | Encephalocele |
| 34 | A2686 | A1783 |  | Male | 21 | Brain | Encephalocele |
| 35 | A2101 | A2694 |  | Male | 18 | Brain | Encephalocele |
| 36 | A1542 | A2777 |  | Male | 19 | Brain | Encephalocele |
| 37 | A2060 | A2928 |  | Male | 20 | Brain | Encephalocele |
| 38 | A1503 | A1818 |  | Female | 26 | Brain | Encephalocele |
| 39 | A1838 | A2918 |  | Male | 15 | Brain | Encephalocele |
