# Supplementary material for: Reduced H3K27me3 leads to abnormal Hox gene expression in neural tube defects
Source: Epigenetics Chromatin. 2019 Dec 19;12:76. doi: 10.1186/s13072-019-0318-1 (PMC6921514; doi:10.1186/s13072-019-0318-1)
Supplement: Supplementary file 10 — Additional file 10: Table S5. RT-qPCR primer sequences. All oligonucleotides were synthesized by Sangon Biotech. [file 13072_2019_318_MOESM10_ESM.docx]

Table S5 RT-qPCR primer sequences

| Gene | Accession | Orientation | Sequence | Product size (bp) |
| --- | --- | --- | --- | --- |
| Nr2e1 | NM_152229.2 | F240 | GTGTGGAGGGAGAGATTGGA | 141 |
|  |  | R380 | AGGCTGAAGAGGGAGGACAG |  |
| Fezf1 | NM_028462.1 | F2021 | TTCCATTGTGTCGTTTCTGC | 140 |
|  |  | R2160 | ATGTTGACCGCACCTTCAC |  |
| Six6 | NM_011384.4 | F835 | AGTGAGTGCGACATCTGAGC | 115 |
|  |  | R949 | GCCGTATTTGCTGGTCTTGT |  |
| Hand1 | NM_008213.2 | F666 | AGGAGAGGAGACGCACAGAG | 129 |
|  |  | R794 | AGGCGATGTAACTGGTAGCC |  |
| Hoxd8 | NM_008276.3 | F1877 | CCTGTGCTCCCGTGTTGTA | 115 |
|  |  | R1991 | GGGCTCATTTCCAATCTGTG |  |
| Abp1 | NM_001161621.1 | F1631 | CCCTAATGGTGTGATGGAGAC | 109 |
|  |  | R1739 | AAGCAGGTGGGTTTGTAAGC |  |
| Hoxb4 | NM_010459.7 | F99 | CCCAACTTCTGAGGGATGC | 124 |
|  |  | R222 | CCTGGCTTTCTCCACAACAG |  |
| Hoxa5 | NM_010453.5 | F205 | AGCCACAAATCAAGCACACA | 127 |
|  |  | R331 | CGCTCACGGAACTATGATCTC |  |
| Hoxc5 | NM_175730.5 | F1718 | GGAGCAGCAAGAATAGGGAAC | 124 |
|  |  | R1841 | CACAGCAAGTGGAAACAACAG |  |
| E030019B13Rik | NR_045082.1 | F235 | ACAAGGTAAGCGGTGTCTGG | 129 |
| 13Rik |  | R363 | AGCAAGTCCATCCCTACGAA |  |
| Ermn | NM_029972.3 | F390 | TCCTCCGAACAGCAGTAACC | 107 |
|  |  | R496 | CCAGCCATTCGATTTCAGTT |  |
| Rax | NM_013833.2 | F1429 | GAAAGAGGCCAAACGGAAAT | 106 |
|  |  | R1534 | GATGATAGGCGCTGATGCTT |  |
| Six3os1 | NR_015385.2 | F816 | TCTGTTTGGAGACACCCAGAA | 121 |
|  |  | R936 | AGCCCTATTCCAGAGCACAA |  |
| Vax1 | NM_009501.1 | F68 | AGAACGCGCACAAGGAGA | 141 |
|  |  | R208 | CCGCTGAGGAATTGGATTTA |  |
| Nkx2-1 | NM_009385.3 | F530 | GTCCTCGGAAAGACAGCATC | 105 |
|  |  | R634 | GTGCTTTGGACTCATCGACA |  |
| Erv3 | NM_001166206.1 | F1205 | CAAATTCCTCGGGTTGAATG | 115 |
|  |  | R1319 | AGCGAGAGAGAAAGGTGGTG |  |
| Prap1 | NM_009475.2 | F251 | GCTTCCTGAACCCAAACAGA | 110 |
|  |  | R360 | CCTGAAGAGGACTGCGAAGA |  |
| Chac1 | NM_026929.4 | F1212 | TCTGTCCACCCAGACTACCC | 124 |
|  |  | R1335 | AGAGGGATGCTGACCCTTG |  |
| Hoxd3 | NM_010468.2 | F1910 | CATAAATCAGCCGCAAGGAT | 111 |
|  |  | R2020 | GGATGGGTCGAGGACTTACC |  |
| Rhox9 | NM_023894.1 | F649 | ATGGATGGGTGTGGATGAAT | 113 |
|  |  | R761 | TCTCTGTAATCGGTGGCAGTT |  |
| Hoxd4 | NM_010469.2 | F440 | GGCCCAGAAAGGTAAATGCT | 116 |
|  |  | R555 | CAGCAGCAGAAAGGGAGTTT |  |
| Hoxb5 | NM_008268.2 | F1666 | CTCTGTGAATCCGTGGGTCT | 121 |
|  |  | R1786 | CCCGCTCACTACAAATGGTC |  |
| Hoxc4 | NM_013553.2 | F1767 | GGGAGCTGTTCAGTTTGAGG | 101 |
|  |  | R1867 | TAACCACGATGAGGGTAGGG |  |
| Nkx2-5 | NM_008700.2 | F1157 | GAGCCTGGTAGGGAAAGAGC | 111 |
|  |  | R1267 | CTGAGGGACAGGGCATAGTG |  |
| Hoxd1 | [NM_010467.2](http://www.ncbi.nlm.nih.gov/entrez/viewer.fcgi?db=nucleotide&id=112983631) | F1050 | AGTCCCATCAAATCTGGCCG | 124 |
|  |  | R1173 | TTCAAAGGTGGGGAGCAGTC |  |
| Hoxa4 | [NM_008265.3](http://www.ncbi.nlm.nih.gov/entrez/viewer.fcgi?db=nucleotide&id=142381148) | F752 | TGCGATCTTCCAACACTGCC | 107 |
|  |  | R858 | AATGGGTGTGGAAGCACCAG |  |
| Utx | NM_009483.2 | F4628 | CCTCCATTACCATCCGCCTC | 189 |
|  |  | R4816 | TACTCTCCCGTCCAGTTGGT |  |
| Suz12 | [NM_199196.2](https://www.ncbi.nlm.nih.gov/entrez/viewer.fcgi?db=nucleotide&id=253314521) | F1866 | GAGAAGTGGAGCAGCAGAGAACATAC | 81 |
|  |  | R1946 | GCCGAAGAGGTAAGCAGGTATCAC |  |
| Ezh2 | [NM_007971.2](https://www.ncbi.nlm.nih.gov/entrez/viewer.fcgi?db=nucleotide&id=226442802) | F905 | GTGCTCTGCCTCCTGAATGT | 138 |
|  |  | R1042 | GGAAGGGATGTAGGAAGCAGTC |  |
| Actb | [NM_007393.4](http://www.ncbi.nlm.nih.gov/entrez/viewer.fcgi?db=nucleotide&id=818213467) | F1086 | AGATCAAGATCATTGCTCCTCCT | 174 |
|  |  | R1259 | ACGCAGCTCAGTAACAGTCC |  |
