# Supplementary material for: Reduced H3K27me3 leads to abnormal Hox gene expression in neural tube defects
Source: Epigenetics Chromatin. 2019 Dec 19;12:76. doi: 10.1186/s13072-019-0318-1 (PMC6921514; doi:10.1186/s13072-019-0318-1)
Supplement: Supplementary file 11 — Additional file 11: Table S6. ChIP-qPCR primer sequences. All oligonucleotides were synthesized by Sangon Biotech. [file 13072_2019_318_MOESM11_ESM.docx]

**Table S6 ChIP-qPCR primer sequences**

| Primer | Orientation | Sequence | Product size (bp) |
| --- | --- | --- | --- |
| Hoxa4 | Forward | GCGACCCGAGCAGTCCC | 253 |
|  | Reverse | ACGCCCGCTGCCCTCC |  |
| Hoxa5 | Forward | CTCTGGAATAAAACGAAGGAGGC | 61 |
|  | Reverse | GGACAAAGAATCAAAGGGCGAG |  |
| Hoxb4 | Forward | GCCTCTAACTTTGTTCACTTGAC | 117 |
|  | Reverse | AGCCATTAATTTCTGGGAATTGC |  |
| Hoxb5 | Forward | TGAGGAAGCTTCACATCAGCCACG | 97 |
|  | Reverse | CCAAGCTTTGCTCGCCCCCAC |  |
| Hoxc4 | Forward | CATCCAACCTGCACTGAAGC | 119 |
|  | Reverse | AGTGCCTTTCCAGTGGTCTC |  |
| Hoxc5 | Forward | GCCAAGCGATGCTACAAGAT | 205 |
|  | Reverse | CGGGAGGTTAGTGATGGAAG |  |
| Hoxd1 | Forward | GAGTAACTTGACCTTCTCAGAG | 179 |
|  | Reverse | ATTGCGGGAGAAAGGCAGGGAAG |  |
| Hoxd3 | Forward | CCAGGCGCGTCCTCC | 51 |
|  | Reverse | GGATCCTTGCGGCTGATTTAT |  |
| Hoxd4 | Forward | GAAGACAAAAGCCGAGATTAC | 188 |
|  | Reverse | TAGGAAGAAAGCATGTTTATCAC |  |
| Hoxd8 | Forward | GGCGAGGCCATCAATCC | 51 |
|  | Reverse | GACCTCGGGTGCAAAATGAC |  |
